# Dietary protein intake and all-cause and cause-specific mortality: results from the Rotterdam Study and a meta-analysis of prospective cohort studies

**DOI:** 10.1007/s10654-020-00607-6

**Published:** 2020-02-19

**Authors:** Zhangling Chen, Marija Glisic, Mingyang Song, Hamid A. Aliahmad, Xiaofang Zhang, Alice C. Moumdjian, Valentina Gonzalez-Jaramillo, Niels van der Schaft, Wichor M. Bramer, Mohammad Arfan Ikram, Trudy Voortman

**Affiliations:** 1 Department of Epidemiology, Erasmus University Medical Center, Rotterdam, The Netherlands; 2 Department of Nutrition, Harvard T. H. Chan School of Public Health, Boston, MA USA; 3 Swiss Paraplegic Research, Nottwil, Switzerland; 4 Department of Epidemiology, Harvard T. H. Chan School of Public Health, Boston, MA USA; 5 Clinical and Translational Epidemiology Unit and Division of Gastroenterology, Massachusetts General Hospital and Harvard Medical School, Boston, MA USA; 6 Medical Library, Erasmus MC University Medical Center, Rotterdam, The Netherlands; 7 Department of Epidemiology, Erasmus MC, Office Na-2718, PO Box 2040, 3000 CA Rotterdam, The Netherlands; 8 Department of Epidemiology, Erasmus MC, Office Na-2716, PO Box 2040, 3000 CA Rotterdam, The Netherlands

**Keywords:** Dietary protein intake, All-cause mortality, Cause-specific mortality, Cardiovascular mortality, Cohort study

## Abstract

Evidence for associations between long-term protein intake with mortality is not consistent. We aimed to examine associations of dietary protein from different sources with all-cause and cause-specific mortality. We followed 7786 participants from three sub-cohorts of the Rotterdam Study, a population-based cohort in the Netherlands. Dietary data were collected using food-frequency questionnaires at baseline (1989–1993, 2000–2001, 2006–2008). Deaths were followed until 2018. Associations were examined using Cox regression. Additionally, we performed a highest versus lowest meta-analysis and a dose–response meta-analysis to summarize results from the Rotterdam Study and previous prospective cohorts. During a median follow-up of 13.0 years, 3589 deaths were documented in the Rotterdam Study. In this cohort, after multivariable adjustment, higher total protein intake was associated with higher all-cause mortality [e.g. highest versus lowest quartile of total protein intake as percentage of energy (Q4 versus Q1), HR = 1.12 (1.01, 1.25)]; mainly explained by higher animal protein intake and CVD mortality [Q4 versus Q1, CVD mortality: 1.28 (1.03, 1.60)]. The association of animal protein intake and CVD was mainly contributed to by protein from meat and dairy. Total plant protein intake was not associated with all-cause or cause-specific mortality, mainly explained by null associations for protein from grains and potatoes; but higher intake of protein from legumes, nuts, vegetables, and fruits was associated with lower risk of all-cause and cause-specific mortality. Findings for total and animal protein intake were corroborated in a meta-analysis of eleven prospective cohort studies including the Rotterdam Study (total 64,306 deaths among 350,452 participants): higher total protein intake was associated with higher all-cause mortality [pooled RR for highest versus lowest quantile 1.05 (1.01, 1.10)]; and for dose–response per 5 energy percent (E%) increment, 1.02 (1.004, 1.04); again mainly driven by an association between animal protein and CVD mortality [highest versus lowest, 1.09 (1.01, 1.18); per 5 E% increment, 1.05 (1.02, 1.09)]. Furthermore, in the meta-analysis a higher plant protein intake was associated with lower all-cause and CVD mortality [e.g. for all-cause mortality, highest versus lowest, 0.93 (0.87, 0.99); per 5 E% increment, 0.87 (0.78, 0.98), for CVD mortality, highest versus lowest 0.86 (0.73, 1.00)]. Evidence from prospective cohort studies to date suggests that total protein intake is positively associated with all-cause mortality, mainly driven by a harmful association of animal protein with CVD mortality. Plant protein intake is inversely associated with all-cause and CVD mortality. Our findings support current dietary recommendations to increase intake of plant protein in place of animal protein.

*Clinical trial registry number and website* NTR6831, https://www.trialregister.nl/trial/6645

**Electronic supplementary material:**

The online version of this article (10.1007/s10654-020-00607-6) contains supplementary material, which is available to authorized users.

## Introduction

Defining the role of dietary protein intake in health has been a long-standing research topic of interest and remains a high priority in nutrition research. Although protein delivers amino acids that are crucial for various body functions, protein intake in the general population tends to be much higher than required [1]. Short-term randomized clinical trials have suggested that consumption of high-protein diets may favor weight management and improve blood lipid and lipoprotein profiles and glycemic regulation [2–4]. However, several prospective observational studies have reported that long-term high intake of total protein and protein from animal-based food sources is associated with higher risk of type 2 diabetes [5] and cardiovascular diseases (CVD) [6].

Recently, to further explore the role of habitual dietary protein intake in overall health, several previous epidemiological prospective cohort studies have examined the associations between protein intake and all-cause and cause-specific mortality [7–15]. Although generally pointing towards harmful or null associations for animal protein and beneficial or null associations for plant protein, the results are not entirely consistent. For example, Song et al. observed in the Nurse Health Study and the Health Professionals Follow-Up Study that higher animal protein intake was associated with higher CVD mortality risk, while higher plant protein intake was associated with lower risk of all-cause and CVD mortality [11]. In contrast, Kelemen et al. [8] and Budhathoki et al. [16], reported null associations of total and animal protein with risk of all-cause and CVD mortality and beneficial association of plant protein with CVD mortality in the Iowa Women’s Health Study and the Japan Public Health Center-based Prospective Cohort, respectively. And Tharrey et al. observed that higher animal protein intake was associated with higher CVD mortality, but plant protein intake was not associated with CVD mortality in the Adventist Health Study-2 [14]. Furthermore, evidence for protein intake from more specific animal food sources (e.g. protein from meat and dairy) and plant food sources (e.g. protein from legumes, nuts, and grains) and mortality, which may partly explain the inconsistencies in the previous studies is more limited [11] and remains to be further clarified. Moreover, to date no meta-analysis that summarizes associations between protein intake and mortality has been published.

Therefore, we aimed to investigate the associations of total protein intake and protein from different food sources with all-cause and cause-specific mortality in the Rotterdam Study. Also, to further clarify the current evidence from previous studies, we systematically reviewed and meta-analyzed our findings with those from previous prospective studies to evaluate the association of dietary protein intake with mortality.

## Methods

The current study consisted of two stages. First, we analyzed the associations of protein intake with all-cause and cause-specific mortality in the Rotterdam study. Second, we conducted a systematic review and meta-analysis by combining the new results from the Rotterdam Study with results from previous prospective cohort studies.

### Methods for the Rotterdam Study analyses

#### Study design and population in the Rotterdam Study

The first stage of this study was conducted within the Rotterdam study, a prospective cohort study of participants aged 45 years and above in Rotterdam, the Netherlands [17]. Briefly, the Rotterdam Study currently consists of three sub-cohorts available. The first sub-cohort of the Rotterdam Study (RS-I) was initiated in the period of 1989–1993 by recruiting participants aged ≥ 55 years from the district of Ommoord (n = 7983). In 2000–2001, the study was extended with a second sub-cohort (RS-II) including new individuals who had become 55 years of age or moved into the study area (n = 3011). In 2006-08, a third sub-cohort (RS-III) was started of new individuals aged 45 years and older (n = 3932). Until the end of 2008, 14,926 participants were contained in the three sub-cohorts at baseline. Information every 3–5 years was collected through interviews, for which the participants were visited at their homes, through questionnaires which the participants returned, and through examinations in our dedicated research center which is in the Ommoord district. In the home interviews, we collected information such as education level, smoking status, and medical history. At the examination center, we mainly focused on examinations of imaging (of heart, blood vessels, eyes, skeleton, and brain) and on collecting biospecimens that enabled further in-depth molecular and genetic analysis. The Rotterdam Study has been approved by the institutional review board (Medical Ethics Committee) of Erasmus Medical Center and by the review board of The Netherlands Ministry of Health, Welfare and Sports. The approval has been renewed every 5 years. All participants gave informed consent [17].

For the current analysis within the Rotterdam Study, of the 14,926 participants who participated at baseline, we had dietary data available for 9701. Main reasons for absence of valid dietary data among the 5225 participants were: no dietary data available (n = 4890) including not having received dietary assessment because of logistic reasons, living in a resident home for elderly or suspected dementia, and not having completed the dietary assessment; or excluded as invalid dietary data (n = 335) defined as an estimated energy intake of < 500 or > 5000 kcal/day) [18, 19]. From these 9701 participants, we further excluded 1914 participants with CVD, diabetes, or cancer at baseline, and 1 participant without follow-up data on mortality, leaving 7786 participants for the main analyses (Supplemental Figure 1).

#### Dietary assessment

Dietary intake in the Rotterdam Study was assessed at baseline in all three sub-cohorts using a semi-quantitative food questionnaire (FFQ) as described in more details elsewhere [19, 20]. Briefly, we used an FFQ with 170 food items to assess dietary intake at baseline of RS-I (1989–1993) and at baseline of RS-II (2000–2001). This 170-item FFQ was validated against fifteen 24-h food records and four 24-h urinary urea excretion samples which were collected on non-consecutive days over a period of a year in a subsample of the Rotterdam Study (n = 80) [21]. Adjusted Pearson’s correlation coefficients between protein intake measured with the FFQ and protein measured with the food records were 0.66 for total protein intake, and 0.59 for plant protein intake; and Spearman’s correlation between protein measured with the FFQ and protein measured from urinary urea was 0.67 for total protein intake. Further details are provided in the study by Klipstein-Grobusch et al. [22]. A 389-item FFQ was used to assess dietary intake at baseline of RS-III (2006–2008). This 389-item FFQ was previously validated in two other Dutch populations using a 9-day dietary record [22] and a 4-week dietary history [23]. Pearson’s correlations for intakes of different nutrients varied from 0.40 to 0.86. Food intake data were converted to energy and nutrient intake based on the Dutch Food Composition tables via which 2152 foods the data on 133 nutrients can be viewed.

#### Ascertainment of death

Information on vital status of the participants was obtained from clinical follow-up data collection and from municipal records. General practitioners reported events of interest by means of a computerized system or notified new events annually. Trained research assistants subsequently collected information from medical records at the general practitioners’ offices, hospitals and nursing homes. Two research physicians independently identified the events according to the International Classification of Diseases, Tenth revision (ICD-10). Afterwards, a senior physician reviewed all coded events. Cause-specific mortality was recoded according to the ICD-10 codes (CVD cause: F01, I05-99 (non-stroke CVD cause: I05-51, 70-99, stroke cause: F01, I60-69.8); cancer cause: C01-97). Coded information on all-cause mortality was available until May 2018 and coded information on cause-specific mortality was available until January 2015.

#### Covariates

Information on age, sex, smoking status, and education level was obtained from questionnaires at baseline. Information on physical activity was obtained using the adapted version of the Zutphen Physical Activity Questionnaire at the third visit of RS-I (1997-99) and at baseline of RS-II, and the LASA Physical Activity Questionnaire at baseline of RS-III. Physical activities were weighted according to intensity with Metabolic Equivalent of Task (MET), from the Compendium of Physical Activities version 2011. To account for differences between the two questionnaires, questionnaire-specific z-scores of MET-hours per week were calculated. We measured body weight and height at baseline in our research center and body mass index (BMI) was calculated. A previously defined diet quality score was calculated to reflect adherence to Dutch dietary guidelines as described in detail elsewhere [20]. Briefly, this was a sum-score of the adherence to guidelines for 14 items, including: vegetables (≥ 200 g/day), fruit (≥ 200 g/day), whole-grains (≥ 90 g/day), legumes (≥ 135 g/week), nuts (≥ 15 g/day), dairy (≥ 350 g/day), fish (≥ 100 g/week), tea (≥ 450 mL/day), ratio whole-grains: total grains (≥ 50%), ratio unsaturated fats and oils: total fats (≥ 50%), red and processed meat (< 300 g/week), sugar-containing beverages (≤ 150 mL/day), alcohol (≤ 10 g/day) and salt (≤ 6 g/day). We scored every participant as adhering to this item (‘yes’ scored as 1) or not adhering to the item (‘no’ scored as 0). The total diet quality score theoretically ranged from 0 (no adherence) through 14 (full adherence). We previously reported that a higher score was associated with a lower premature mortality risk and a lower risk of developing some of the chronic diseases on which the guidelines were based [20]. Information on CVD, diabetes, and cancers was obtained from general practitioners, pharmacies’ datasets, Nationwide Medical Register, or follow-up examinations in our research center.

#### Data analyses

We expressed intake of dietary protein and other macronutrients as a percentage of total energy consumption. Baseline characteristics of the Rotterdam population are presented for the whole group and by quartiles of total, animal, or plant protein intake. Trends of these characteristics across quartiles of protein intake were examined by using means and linear regression for continuous variables, or Chi square tests for categorical variables. After confirming that the assumption for proportional hazards was met on the basis of Schoenfeld residuals [24] and Martingales residuals [25], we used Cox proportional hazard models to analyze associations of dietary protein intake with all-cause and cause-specific mortality. Non-linearity of associations of protein intake with all outcomes were explored using natural cubic splines (degrees of freedom = 3). Because effects of macronutrient intake cannot be separated from the effects of overall energy intake or intake of other macronutrients, we modelled macronutrient substitution effects. For our main models, we used multivariable nutrient density substitution models for protein intake at the expense of carbohydrate. For this aim, models were used with adjustment for total energy intake and percentage of energy from subtypes of fats (saturated fat (SFA), monounsaturated fat (MUFA), polyunsaturated fat (PUFA), and trans-fat (TSF)), and from alcohol [26]. Therefore, coefficients from these models were interpreted as the estimated effects of substituting a certain percentage of energy from protein intake for equivalent energy from carbohydrate intake. We estimated hazard ratios (HRs) and their 95% confidence intervals (CIs) of mortality by comparing participants in each quartile of protein intake as percentage of energy with those in the lowest quartile. To quantify a linear trend, we assigned the median within each quartile and modeled this variable continuously. Furthermore, we also modelled dietary protein intake as continuous variable and estimated HRs and 95%CIs per 5 energy percent (E%) increment from protein at the expense of carbohydrate.

For all main analyses, we included intake of protein, SFA, MUFA, PUFA, TSF, total energy, alcohol, baseline age, sex, and RS-cohort in model 1; we additionally adjusted for smoking status, education level, overall diet quality score, fiber intake, physical activity, and BMI in model 2. For analysis of animal and plant protein intake, mutual adjustment for plant and animal protein was performed.

#### Additional analyses

We further examined if the associations of animal protein intake and plant protein intake with all-cause and cause-specific mortality differed by protein from specific animal and plant food sources, such as protein from meat, dairy, fish, and eggs, and protein from grains, potatoes, legumes, nuts, vegetables, and fruits. In this modelling approach, protein from meat, dairy, fish and eggs was entered in model per 5 E%, and protein from grains, potatoes, and legumes, nuts, vegetables, and fruits were entered in model per 3 E%. The percentages of energy intake from protein from meat, dairy, fish, and eggs and protein from grains, potatoes, and legumes, nuts, vegetables, and fruits were simultaneously included in one model, with adjustment for all other covariates in model 2.

#### Sensitivity analyses

We conducted a series of sensitivity analyses to test robustness of our main results. First, we replaced fat intake by carbohydrate in the main models (model 2), to examine whether it made a difference if dietary protein was consumed at the expense of fat instead of carbohydrate. Second, we examined the interaction effect of total, animal, or plant protein with age, sex, BMI, or physical activity by including their interaction terms in main models (model 2), to explore whether the associations of protein intake and mortality differed by these factors. Third, we adjusted for the modified diet quality score without main protein food sources (e.g. without red and processed meat, fish, and legumes) in the analyses. Last, to minimize reverse causality bias, we excluded the participants who died within the first 2 years of follow-up in the Rotterdam study.

We performed all analyses based on combined data from RS-I, RS-II, and RS-III. All variables included in analyses were used to predict missingness patterns. Missing values on covariates were assumed to be missing at random and accounted for using multiple imputations (m = 10 imputations) [27]. Supplemental Table 1 shows the percentage of missing data for covariates in the Rotterdam Study. Statistical procedures were performed with the use of R version 3.3.1 (The R Foundation for Statistical Computing, Vienna, Austria).

### Methods for the systematic review and meta-analysis

#### Data sources, searches, extraction, and quality assessment

The systematic review was conducted using a predefined protocol and reported in accordance with the PRISMA and MOOSE guidelines [28, 29]. Medline (Ovid), Embase.com, and the Cochrane Central Register of Controlled Trials were searched from inception until August 27, 2019 (date last searched), with assistance of an experienced biomedical information specialist. The detailed search strategy is shown in Supplemental Table 2. Two independent reviewers conducted an initial screening of all titles or abstracts and then evaluated all potentially relevant articles based on full text reviews. Eligible studies were included if they (1) were observational studies with a longitudinal design (i.e., prospective cohort); and (2) had assessed the variance of estimates of the association between dietary protein intake (total, animal and/or plant protein) with all-cause mortality and/or cause-specific mortality in a general population (i.e., populations that were not selected based on pre-existing health conditions). We contacted the investigators for relevant data if their studies were potentially eligible for this study. We extracted the following characteristics from the included studies: first author, cohort name, country, publication year, age at entry, sex, sample size, duration of follow-up, assessment of dietary protein intake, ascertainment of outcomes, the most adjusted association estimates and corresponding measures of variation, and variables that were entered into the multivariable model as potential confounders. In case of multiple publications from the same study, we included the most up-to date or comprehensive information. We used the nine-star Newcastle–Ottawa Scale (NOS) to assess study quality based on selection of three domains: selection of participants (population representativeness), comparability (adjustment for confounders), and ascertainment of outcomes of interest. Nine points on the NOS reflects the highest study quality [30].

#### Data synthesis and analysis

We conducted highest versus lowest and dose–response meta-analyses, using the most adjusted association estimation from each original study. For the main meta-analysis, we estimated pooled RRs for highest versus lowest quantile of protein intake using random-effects models [31]. Heterogeneity was determined using the Cochrane χ^2^ statistic and the I^2^ statistic [32]. We additionally conducted dose–response meta-analyses for all-cause, CVD, and cancer mortality, using a generalized least-squares regression approach [33]. In estimating dose–response trends, several approximations across categories of dietary protein intake were applied: the midpoint or mean value of the amount of dietary protein intake, distributions of deaths and person years, HR and 95% CI. When sufficient data (n ≥ 5) studies [34] contributed to a dose–response meta-analysis, non-linearity was explored using restricted cubic splines with three knots (10%, 50%, and 90%) [35]. A Wald-type test was used to test statistical significance of non-linearity [35].

In the main meta-analyses comparing quantiles, we conducted subgroup analyses stratified by geographical study location. As sensitivity analysis, we examined the influence of individual studies on the overall risk estimates comparing quantiles by recalculating the pooled estimates after excluding one study at a time. As a second set of sensitivity analyses, we additionally incorporated studies reporting estimations not expressed in E% in the dose–response meta-analysis, for which we could only approximate protein intake in E% [14]. Additionally, publication bias was evaluated through a funnel plot [36] and Egger’s test [37, 38]. We used STATA release 12 (Stata Corp, College Station, Texas) for all highest versus lowest meta-analyses. The dose–response meta-analysis was conducted with “dosresmeta” package [35] in R version 3.3.1 (The R Foundation for Statistical Computing, Vienna, Austria).

## Results

### Results in the Rotterdam Study

#### Characteristics of population

For the 7786 participants of the Rotterdam Study in our current analysis, mean age at baseline was 63.7 ± 8.7 years, and 60.8% of all participants were female. Furthermore, average total protein intake was 85.8 ± 25.1 g/day (16.4% ± 2.3% of total energy), this corresponded to 1.20 ± 0.3 g/kg BW/day, which is higher than the recommended intake of 0.8 g/kg BW/day [39]. Most protein came from animal sources (53.6 ± 19.0 g/day, and 10.3% ± 2.5% of total energy). Compared to participants in the lowest quartile of animal protein intake, those in the highest quartile of animal protein had a slightly higher BMI, were more often lower educated, and more likely to smoke. In contrast, compared to the participants in the lowest quartile of plant protein intake, those in the highest quartile of plant protein intake had higher overall diet quality, and were more often highly educated, and less likely to smoke (Table 1, Supplemental Table 3).

**Table 1:** Characteristics of the Rotterdam Study population (n = 7786)

	(n = 7786)	By extreme quartiles of total protein		By extreme quartiles of animal protein		By extreme quartiles of plant protein	
		Quartile 1 (n = 1947) ≤ 14.4 E%	Quartile 4 (n = 1947) > 18.1 E%	Quartile 1 (n = 1947) ≤8.4 E%	Quartile 4 (n = 1947) > 12.1 E%	Quartile 1 (n = 1947) ≤ 5.2 E%	Quartile 4 (n = 1947) > 6.7 E%
Age (years)	63.7 (8.7)	63.9 (9.4)	64.2 (8.2)	62.1 (9.1)	64.9 (8.2)*	66.2 (8.8)	60.6 (7.9)*
Sex (%)							
Female	60.8	12.8	18.4*	12.9	17.9*	13.9	15.6*
Male	39.2	12.7	6.6*	12.1	7.1*	11.1	9.4*
BMI (kg/m^2^)	26.6 (3.9)	25.8 (3.6)	27.5 (4.3)*	25.8 (3.7)	27.4 (4.2)*	26.4 (3.7)	26.6 (4.1)
Smoking status (%)							
Never	23.8	7.2	9.3	7.8	8.9	6.8	9.1
Ever	42.3	11.0	10.1	11.4	9.8	9.6	11.2
Current	23.8	6.7	5.4*	5.7	6.2*	8.5	4.7*
Education level (%)							
Primary	15.3	3.6	4.1	3.1	4.5	4.6	3.1
Low	41.1	10.0	10.9	9.5	10.9	10.8	9.6
Intermediate	27.2	7.2	6.6	7.1	6.4	6.6	6.5
High	15.8	4.1	3.2	5.2	3.1*	2.9	5.7*
Physical activity (MET-hours/week)							
RS-I and II	80.4 (55.7113.2)	74.3 (47.8, 105.6)	83.9 (59.8, 117.5)*	76.7 (49.5108.7)	83.0 (58.1, 116.0)*	75.9 (47.6, 105.5)	84.8 (61.6, 121.0)*
RS-III	43.0 (17.7, 82.6)	41.0 (17.1,84.3)	39.4 (15.0, 56.4)	42.8 (18.0,84.1)	38.0 (15.0, 72.4)	38.0 (16.0, 72.4)	48.0 (18.7, 87.8)*
Dietary intake							
Total protein (g/day)	85.8 (25.1)	76.6 (21.2)	90.2 (25.4)*	78.9 (23.4)	90.4 (25.5)*	83.5 (25.0)	86.2 (24.3)*
Total protein (E%)	16.4 (2.3)	13.0 (1.3)	20.3 (2.1)*	13.4 (1.7)	20.0 (2.3)*	15.8 (3.2)	16.8 (2.9)*
Animal protein(g/day)	53.6 (19.0)	42.9 (14.0)	63.0 (30.0)*	40.5 (13.3)	65.5 (20.9)*	59.4 (21.6)	46.2 (15.8)*
Animal protein (E%)	10.3 (2.5)	7.3 (1.6)	14.2 (2.5)*	6.9 (1.3)	14.5 (2.2)*	11.3 (3.3)	9.1 (3.0)*
Protein from meat (g/day)	21.8 (11.7)	18.2 (9.5)	25.0 (14.6)*	16.7 (9.4)	26.0 (14.7)*	24.4 (13.9)	18.5 (10.5)*
Protein from meat (E%)	4.3 (2.1)	3.1 (1.4)	5.6 (2.6)*	2.8 (1.3)	5.8 (2.6)*	4.7 (2.4)	3.7 (2.0)*
Protein from dairy (g/day)	22.0 (12.3)	17.5 (9.3)	26.6 (14.6)*	16.6 (8.8)	27.9 (15.3)*	24.9 (15.2)	19.0 (10.3)*
Protein from dairy (E%)	4.3 (2.3)	3.0 (1.4)	6.0 (2.8)*	2.8 (1.3)	6.1 (2.8)*	4.7 (2.5)	3.8 (2.1)*
Protein from fish (g/day)	4.0 (4.7)	3.2 (3.8)	4.7 (5.7)*	3.5 (3.8)	4.6 (5.7)*	3.9 (4.8)	4.2 (4.5)
Protein from fish (E%)	0.8 (0.9)	0.5 (0.6)	1.1 (1.2)	0.6 (0.6)	1.0 (1.2)*	0.7 (1.0)	0.8 (0.9)
Protein from eggs (g/day)	1.9 (1.5)	1.8 (1.4)	1.8 (1.4)	1.7 (1.5)	1.9 (1.5)	2.1 (1.6)	1.7 (1.4)
Protein from eggs (E%)	0.4 (0.3)	0.3 (0.2)	0.4 (0.3)	0.3 (0.3)	0.4 (0.3)	0.4 (0.3)	0.3 (0.3)
Plant protein (g/day)	30.3 (8.9)	33.7 (12.1)	27.2 (9.4)*	38.4 (13.7)	25.0 (7.6)*	24.0 (7.1)	39.9 (13.5)*
Plant protein (E%)	6.2 (1.5)	5.7 (1.3)	6.1 (1.5)*	6.5 (1.6)	5.6 (1.2)*	4.5 (0.6)	7.6 (1.1)*
Protein from grains (g/day)	16.0 (7.6)	17.1 (8.1)	13.8 (6.3)*	20.0 (9.3)	12.6 (5.5)*	11.9 (5.1)	20.5 (9.4)*
Protein from grains (E%)	3.1 (1.12)	2.9 (1.1)	3.1 (1.2)	3.3 (1.2)	2.8 (1.1)*	2.2 (0.8)	3.9 (1.2)*
Protein from potatoes (g/day)	2.3 (1.4)	2.5 (1.5)	2.0 (1.3)*	2.3 (1.5)	2.0 (1.2)*	2.3 (1.3)	2.1 (1.5)
Protein from potatoes (E%)	0.4 (0.3)	0.4 (0.3)	0.5(0.3)	0.4 (0.3)	0.5 (0.3)	0.4 (0.2)	0.4 (0.3)
Protein from legumes, nuts, vegetables, and fruits (g/day)	10.1 (8.7)	10.7 (9.3)	8.9 (7.9)*	14.0 (11.3)	7.6 (6.5)*	6.2 (5.0)	16.0 (11.5)*
Protein from legumes, nuts, vegetables, and fruits (E%)	1.90 (1.5)	1.8 (1.4)	2.0 (1.7)*	2.4 (1.8)	1.7 (1.4)*	1.2 (1.0)	3.0 (1.9)*
Total fat (g/day)	82.7 (29.7)	93.5 (34.2)	68.9 (24.2)*	72.8 (27.0)	90.9 (35.1)*	91.2 (33.3)	76.5 (29.7)*
Total fat (E%)	35.3 (6.6)	35.3 (7.3)	34.5 (6.2)*	34.3 (7.3)	35.6 (6.5)*	38.1 (7.1)	32.3 (6.0)*
SFA (g/day)	31.7 (12.3)	35.1 (13.6)	27.0 (10.7)*	29. 2 (12.5)	32.8 (13.4)*	37.1 (14.4)	26.8 (10.5)*
SFA (E%)	13.6 (3.3)	13.3 (3.4)	13.5 (3.3)*	12.4 (3.3)	14.2 (3.4)*	15.5 (3.5)	11.5 (2.6)*
MUFA (g/day)	27.8 (11.2)	31.8 (13.2)	23.0 (8.6)*	24.3 (13.7)	30.9 (9.4)*	30.9 (12.7)	25.9 (11.2)*
MUFA (E%)	11.8 (2.8)	11.9 (3.2)	11.5 (2.5)*	11.5 (3.2)	11.9 (2.7)*	12.8 (3.2)	11.0 (2.7)*
PUFA (g/day)	16.5 (7.8)	19.3 (9.3)	13.0 (6.0)*	19.8 (9.4)	13.1 (6.2)*	16.4 (8.5)	17.0 (8.1)*
PUFA (E%)	7.0 (2.5)	7.2 (2.7)	6.5 (2.5)*	7.4 (2.7)	6.5 (2.5)*	6.8 (2.7)	7.2 (2.3)*
TSF (g/day)	1.7 (1.2, 2.4)	1.9 (1.3, 2.9)	1.4 (1.0, 1.9)	1.6 (1.1, 2.4)	1.5 (1.1, 2.1)*	2.1 (0.25, 3.21)	1.27 (0.91, 1.80)*
TSF (E%)	0.72 (0.52, 1.05)	0.72 (0.52, 1.10)	0.72 (0.54, 0.97)	0.61 (0.45, 0.92)	0.77 (0.59, 1.06)*	0.92 (0.65, 1.33)	0.56 (0.43, 0.74)*
Carbohydrate (g/day)	228.2 (76.6)	269.0 (86.5)	186.2 (56.2)*	275.0 (87.1)	183.9 (56.3)*	216.4 (77.7)	243.7 (80.9)*
Carbohydrate (E%)	43.5 (7.1)	45.5 (7.9)	41.9 (6.7)*	46.7 (7.6)	40.6 (6.8)*	40.1 (7.9)	46.6 (6.4)*
Diet quality score	6.7 (1.9)	6.3 (2.0)	7.2 (1.8)*	6. 8 (2.0)	6.9 (1.8)	5.7 (1.7)	7.7 (1.7)*
Fiber (g)	19.5 (15.1, 26.6)	20.8 (15.4, 28.9)	17.7(14.3, 22.6)*	24.6 (18.1, 33.9)	16.6 (13.6, 20.9)*	15.2 (12.2, 19.5)	26.2 (19.6, 35.2)*

Variables expressed as mean (SD), median (25th percentile–75th percentile), or percentage

*MET* metabolic equivalent of task, E% energy percent, *SFA* saturated fat acids, *MUFA* monounsaturated fat acids, *PUFA* polyunsaturated fat acids, *TSF* trans fat acid

*P*-trend was assessed were tested with linear regression (continuous variables) or with Chi square test (categorical variables). *P < 0.05 for trend across quartiles

#### Associations of protein intake with all-cause mortality and cause-specific mortality

During a median follow-up of 13.0 years (IQR 8.3–19.1 years) for all-cause mortality (data available until May 2018), we documented 3589 deaths. During a median follow-up of 12.9 years (25th–75th percentile, 8.3–19.0 years) for cause-specific mortality (data available until January 2015), we documented 877 CVD deaths (of which, 594 non-stroke CVD deaths and 283 stroke deaths), 896 cancer deaths, and 1289 deaths due to other causes (which consisted of various specific causes, all with relatively small numbers).

As shown in Table 2, in multivariable models (Model 2), higher total protein intake was associated with higher risk of all-cause mortality, CVD mortality, and non-stroke CVD mortality [e.g. for all-cause mortality, the highest quartile versus the lowest quartile of total protein intake as percentage of energy (Q4 versus Q1), HR: 1.12, 95%CI (1.01, 1.25); per 5 E% increment in total protein, HR: 1.09, 95%CI (1.02, 1.17); and for CVD mortality, per 5 E% HR: 1.20, 95%CI (1.05, 1.37)]. These associations were mainly explained by animal protein intake (Table 3) [e.g. Q4 versus Q1, for all-cause mortality, 1.18 (1.05, 1.31), for CVD mortality, 1.28 (1.03, 1.60); per 5 E% increment, for all-cause mortality, 1.20 (1.05, 1.37); for CVD mortality, 1.19 (1.04, 1.37)]. Total, or animal protein intake was not associated with stroke mortality, cancer mortality, and other mortality. Plant protein intake was not associated with all-cause and cause-specific mortality (Table 4).

**Table 2:** Associations of total protein intake with all-cause and cause-specific mortality in the Rotterdam Study (n = 7786, comparison is isocaloric substitution for carbohydrate)

Total protein	HR (95% CI) per 5 E% increment	Quartile 1	Quartile 2	Quartile 3	Quartile 4	*P* trend
	n = 7786	n = 1947	n = 1946	n = 1946	n = 1947	
Median intake (E%)	16.2	13.3	15.3	17.0	19.7	
All-cause mortality						
Number of deaths	n = 3589	n = 878	n = 853	n = 884	n = 974	
Model 1	1.04 (0.97, 1.11)	1 (Reference)	1.00 (0.91, 1.10)	0.96 (0.87, 1.05)	1.04 (0.93, 1.15)	0.60
Model 2	1.09 (1.02, 1.17)	1 (Reference)	1.05 (0.95, 1.16)	1.02 (0.92, 1.13)	1.12 (1.01, 1.25)	0.06
Cardiovascular mortality						
Number of deaths	n = 877	n = 220	n = 191	n = 205	n = 261	
Model 1	1.16 (1.01, 1.32)	1 (Reference)	0.95 (0.78, 1.16)	0.93 (0.76, 1.14)	1.16 (0.94, 1.42)	0.15
Model 2	1.20 (1.05, 1.37)	1 (Reference)	0.99 (0.81, 1.21)	0.99 (0.80, 1.21)	1.22 (0.99, 1.52)	0.06
Non-stroke CVD mortality						
Number of deaths	n = 594	n = 147	n = 125	n = 143	n = 179	
Model 1	1.24 (1.06, 1.45)	1 (Reference)	0.93 (0.73, 1.18)	0.96 (0.76, 1.23)	1.19 (0.92, 1.53)	0.13
Model 2	1.27 (1.08, 1.49)	1 (Reference)	0.96 (0.75, 1.23)	1.02 (0.79, 1.31)	1.23 (0.95, 1.60)	0.04
Stroke mortality						
Number of deaths	n = 283	n = 73	n = 66	n = 62	n = 82	
Model 1	0.99 (0.78, 1.24)	1 (Reference)	1.00 (0.71, 1.40)	0.85 (0.59, 1.21)	1.09 (0.76, 1.57)	0.86
Model 2	1.05 (0.83, 1.33)	1 (Reference)	1.04 (0.74, 1.47)	0.91 (0.64, 1.32)	1.19 (0.82, 1.74)	0.42
Cancer mortality						
Number of deaths	n = 896	n = 243	n = 220	n = 220	n = 213	
Model 1	0.92 (0.81, 1.06)	1 (Reference)	0.92 (0.76, 1.10)	0.87 (0.71, 1.05)	0.84 (0.68, 1.04)	0.10
Model 2	0.94 (0.82, 1.08)	1 (Reference)	0.95 (0.78, 1.14)	0.89 (0.73, 1.08)	0.87 (0.70, 1.08)	0.18
Other mortality						
Number of deaths	n = 1289	n = 311	n = 309	n = 318	n = 351	
Model 1	1.00 (0.90, 1.11)	1 (Reference)	1.04 (0.89, 1.22)	0.95 (0.80, 1.12)	1.02 (0.86, 1.22)	0.99
Model 2	1.09 (0.97, 1.22)	1 (Reference)	1.12 (0.95, 1.32)	1.06 (0.89, 1.25)	1.16 (0.97, 1.39)	0.17

Effect estimates are hazard ratios (HRs) and 95%-confidence intervals (95%CIs) derived from Cox proportional hazards regression models. Estimates are based on pooled results of imputed data

Model 1: age, sex, RS-cohort (RS-I, -II, and -III), intake of total energy, SFA (E%), MUFA (E%), PUFA (E%), TSFA (E%), and alcohol (E%)

Model 2: Model 1 + fiber, overall diet quality score, physical activity (z-score of metabolic equivalents of task-hours/week), education level (primary, lower, intermediate, and high), smoking status (never, ever, current), and BMI

*SFA* saturated fat acids, *MUFA* monounsaturated fat acids, *PUFA* polyunsaturated fat acids, *TSF* trans fat acids, *BMI* body mass index

**Table 3:** Associations of animal protein with all-cause and cause-specific mortality in the Rotterdam Study (n = 7786, comparison is isocaloric substitution for carbohydrate)

Animal protein	HR (95% CI) per 5 E% increment	Quartile 1	Quartile 2	Quartile 3	Quartile 4	*P* trend
	n = 7786	n = 1947	n = 1946	n = 1946	n = 1947	
Median intake (E%)	10.2	7.2	9.3	11.1	13.9	
All-cause mortality						
Number of deaths	n = 3589	n = 692	n = 887	n = 970	n = 1040	
Model 1	1.10 (0.96, 1.25)	1 (Reference)	1.07 (0.96, 1.18)	1.05 (0.95, 1.16)	1.13 (1.01, 1.26)	0.04
Model 2	1.20 (1.05, 1.37)	1 (Reference)	1.06 (0.95, 1.17)	1.08 (0.97, 1.20)	1.18 (1.05, 1.31)	0.003
Cardiovascular mortality						
Number of deaths	n = 877	n = 169	n = 216	n = 219	n = 273	
Model 1	1.16 (1.02, 1.32)	1 (Reference)	1.08 (0.88, 1.32)	0.97 (0.79, 1.20)	1.24 (1.00, 1.54)	0.08
Model 2	1.19 (1.04, 1.37)	1 (Reference)	1.07 (0.87, 1.32)	1.01 (0.82, 1.25)	1.28 (1.03, 1.60)	0.03
Non-stroke CVD mortality						
Number of deaths	n = 594	n = 109	n = 145	n = 150	n = 190	
Model 1	1.25 (1.07, 1.47)	1 (Reference)	1.11 (0.86, 1.43)	1.05 (0.81, 1.35)	1.36 (1.04, 1.77)	0.02
Model 2	1.27 (1.08, 1.49)	1 (Reference)	1.10 (0.85, 1.42)	1.06 (0.81, 1.37)	1.34 (1.03, 1.75)	0.03
Stroke mortality						
Number of deaths	n = 283	n = 60	n = 71	n = 69	n = 83	
Model 1	0.98 (0.78, 1.24)	1 (Reference)	1.01 (0.71, 1.43)	0.84 (0.58, 1.21)	1.03 (0.71, 1.49)	0.99
Model 2	1.05 (0.83, 1.33)	1 (Reference)	1.01 (0.71, 1.44)	0.90 (0.62, 1.30)	1.12 (0.76, 1.64)	0.64
Cancer mortality						
Number of deaths	n = 896	n = 184	n = 248	n = 230	n = 234	
Model 1	0.93 (0.82, 1.07)	1 (Reference)	1.09 (0.90, 1.32)	0.94 (0.77, 1.15)	0.97 (0.78, 1.21)	0.51
Model 2	0.95 (0.82, 1.08)	1 (Reference)	1.08 (0.89, 1.32)	0.94 (0.77, 1.16)	0.98 (0.78, 1.22)	0.54
Other mortality						
Number of deaths	n = 1289	n = 240	n = 301	n = 364	n = 384	
Model 1	1.02 (0.92, 1.14)	1 (Reference)	1.02 (0.86, 1.21)	1.03 (0.87, 1.22)	1.09 (0.91,1.31)	0.22
Model 2	1.09 (0.97, 1.22)	1 (Reference)	1.03 (0.86, 1.22)	1.11 (0.93, 1.32)	1.21 (1.00, 1.46)	0.07

Effect estimates are hazard ratios (HRs) and 95%-confidence intervals (95%CIs) derived from Cox proportional hazards regression models. Estimates are based on pooled results of imputed data

Model 1: age, sex, and RS-cohort (RS-I, -II, and –III), total energy, plant protein (E%), SFA (E%), MUFA (E%), PUFA (E%), TSFA (E%), and alcohol (E%)

Model 2: Model 1 + fiber, overall diet quality score, physical activity (z-score of metabolic equivalents of task-hours/week), education level (primary, lower, intermediate, and high), smoking status (never, ever, current), and BMI

*SFA* saturated fat acids, *MUFA* monounsaturated fat acids, *PUFA* polyunsaturated fat acids, *TSF* trans fat acids, *BMI* body mass index

**Table 4:** Associations of plant protein with all-cause and cause-specific mortality in the Rotterdam study (n = 7786, comparison is isocaloric substitution for carbohydrate)

Plant protein	HR (95% CI) per 5 E% increment	Quartile 1	Quartile 2	Quartile 3	Quartile 4	*P* trend
	n = 7786	n = 1947	n = 1946	n = 1946	n = 1947	
Median intake (% energy)	5.8	4.6	5.5	6.2	7.3	
All-cause mortality						
Number of deaths	n = 3589	n = 1176	n = 986	n = 813	n = 614	
Model 1	0.80 (0.67, 0.95)	1 (Reference)	0.85 (0.78, 0.93)	0.84 (0.76, 0.93)	0.90 (0.80, 1.01)	0.05
Model 2	1.09 (0.88, 1.35)	1 (Reference)	0.93 (0.85, 1.03)	0.94 (0.84, 1.04)	1.06 (0.92, 1.21)	0.53
Cardiovascular mortality						
Number of deaths	n = 877	n = 282	n = 235	n = 210	n = 150	
Model 1	1.02 (0.72, 1.46)	1 (Reference)	0.88 (0.74, 1.06)	0.98 (0.80, 1.19)	1.03 (0.81, 1.30)	0.72
Model 2	1.28 (0.84,1.96)	1 (Reference)	0.96 (0.80, 1.17)	1.06 (0.86, 1.31)	1.19 (0.91, 1.57)	0.17
Non-stroke CVD mortality						
Number of deaths	n = 594	n = 190	n = 155	n = 148	n = 101	
Model 1	1.03 (0.67, 1.59)	1 (Reference)	0.85 (0.68, 1.05)	0.99 (0.78, 1,25)	0.98 (0.74, 1.31)	0.91
Model 2	1.32 (0.79, 2.22)	1 (Reference)	0.92 (0.73, 1.17)	1.07 (0.83, 1.39)	1.16 (0.83, 1.62)	0.29
Stroke mortality						
Number of deaths	n = 283	n = 92	n = 80	n = 62	n = 49	
Model 1	1.09 (0.55, 2.16)	1 (Reference)	0.96 (0.70, 1.31)	0.96 (0.68, 1.37)	1.13 (0.74,1.71)	0.64
Model 2	1.36 (0.61, 3.03)	1 (Reference)	1.05 (0.76, 1.46)	1.05 (0.72, 1.52)	1.27 (0.79, 2.04)	0.37
Cancer mortality						
Number of deaths	n = 896	n = 305	n = 227	n = 204	n = 160	
Model 1	0.72 (0.48, 1.04)	1 (Reference)	0.76 (0.64, 0.91)	0.80 (0.66, 0.97)	0.82 (0.65, 1.03)	0.08
Model 2	0.84 (0.53, 1.32)	1 (Reference)	0.81 (0.68, 0.98)	0.85 (0.69, 1.04)	0.90 (0.69, 1.17)	0.44
Other mortality						
Number of deaths	n = 1289	n = 441	n = 392	n = 261	n = 195	
Model 1	0.59 (0.43, 0.83)	1 (Reference)	0.92 (0.80, 1.07)	0.75 (0.64, 0.89)	0.85 (0.69, 1.03)	0.01
Model 2	0.90 (0.62, 1.3)	1 (Reference)	1.04 (0.89, 1.21)	0.87 (0.73, 1.04)	1.06 (0.84, 1.34)	0.87

Effect estimates are hazard ratios (HRs) and 95%-confidence intervals (95%CIs) derived from Cox proportional hazards regression models. Estimates are based on pooled results of imputed data

Model 1: age, sex, and RS-cohort (RS-I, -II, and -III), total energy, animal protein (E%), SFA (E%), MUFA (E%), PUFA (E%), TSFA (E%), and alcohol (E%)

Model 2: Model 1 + fiber, overall diet quality score, physical activity (z-score of metabolic equivalents of task-hours/week), education level (primary, lower, intermediate, and high), smoking status (never, ever, current), and BMI

*SFA* saturated fat acids, *MUFA* monounsaturated fat acids, *PUFA* polyunsaturated fat acids, *TSF* trans fat acids, *BMI* body mass index

We further examined associations of protein intake from more specific animal and plant food sources with mortality. As shown in Table 5, higher intake of protein from meat and dairy was associated with higher all-cause mortality, CVD mortality, non-stroke CVD mortality, and other mortality [e.g. for CVD mortality, per 5 E% protein from meat: 1.32 (1.12, 1.57), protein from dairy: 1.27 (1.09, 1.49)], although not with stroke mortality and cancer mortality. Protein intake from fish and eggs was not associated with all-cause and cause-specific mortality. For protein intake from more specific plant food sources, higher intake of protein from grains and potatoes was not associated with all-cause and cause-specific mortality [e.g. for CVD mortality, per 3 E% protein from grains: 1.11 (0.87, 1.40)], but higher intake of protein from legumes, nuts, vegetables, and fruits was associated with lower risk of all-cause, CVD, non-stroke CVD, stroke, cancer, and other mortality [e.g. for CVD mortality, per 3 E% protein from legumes, nuts, vegetables, and fruits, 0.75 (0.58, 0.97)].

**Table 5:** Associations of protein intake from various foods with all-cause and cause-specific mortality in the Rotterdam Study (n = 7786, comparison isocaloric substitution for carbohydrate)

	All-cause mortality	CVD mortality	Non-stroke CVD mortality	Stroke mortality	Cancer mortality	Other mortality
	HR (95%CI)	HR (95%CI)	HR (95%CI)	HR (95%CI)	HR (95%CI)	HR (95%CI)
Protein intake (per 5 E%)						
From meat	1.16 (1.07, 1.27)	1.32 (1.12, 1.57)	1.35 (1.10, 1.66)	1.27 (0.94, 1.71)	1.03 (0.87, 1.22)	1.23 (1.07, 1.42)
From dairy	1.19 (1.10, 1.28)	1.27 (1.09, 1.49)	1.35 (1.12, 1.64)	1.12 (0.85, 1.48)	1.15 (0.98, 1.34)	1.18 (1.04, 1.34)
From fish	0.83 (0.66, 1.05)	0.99 (0.62, 1.58)	1.22 (0.70, 2.11)	0.60 (0.25, 1.46)	0.88 (0.56, 1.38)	0.75 (0.50, 1.12)
From eggs	0.78 (0.41, 1.47)	1.49 (0.41, 5.32)	2.01 (0.44, 9.22)	0.75 (0.08, 7.52)	0.50 (0.14, 1.82)	0.57 (0.19, 1.70)
Protein intake (per 3 E%)						
From grains	1.04 (0.94, 1.16)	1.11 (0.87, 1.40)	1.13 (0.85, 1.50)	1.08 (0.71, 1.63)	0.92 (0.74, 1.15)	1.11 (0.91, 1.34)
From potatoes	0.86 (0.58, 1.27)	1.01 (0.997, 1.01)	2.26 (0.95, 5.37)	1.00 (0.98, 1.01)	1.00 (0.99, 1.01)	1.00 (0.99, 1.00)
From legumes, nuts, vegetables, and fruits	0.75 (0.67, 0.85)	0.75 (0.58, 0.97)	0.70 (0.50, 0.96)	0.86 (0.56, 1.31)	0.72 (0.57, 0.92)	0.64 (0.50, 0.80)

Effect estimates are hazard ratios (HRs) and 95%-confidence intervals (95%CIs) derived from Cox proportional hazards regression models with adjustment for SFA (E%), MUFA (E%), PUFA (E%), TSF (E%), total energy, alcohol (E%), fiber, age, sex, RS-cohorts (RS-I, -II, and -III), education level(primary, lower, intermediate, and high), smoking status (never, ever, current), physical activity (z-score of metabolic equivalents of task-hours/week), diet quality score, and BMI. Protein from meat, fish, dairy, and eggs and protein from grains, potatoes, and legumes, nuts and vegetables are mutually adjusted. Estimates are based on pooled results of imputed data

*SFA* saturated fat acids, *MUFA* monounsaturated fat acids, *PUFA* polyunsaturated fat acids, *TSF* trans fat acids, *BMI* body mass index

#### Sensitivity analyses

In the Rotterdam Study, we observed similar results for protein intake and all-cause and cause-specific mortality when at the expense of fat instead of carbohydrate (Supplemental Table 4). We also observed no interaction effects of protein intake with age, BMI, or physical activity, but we did observe a significant interaction effect of animal protein intake with sex for all-cause mortality (*P* value for the interaction term = 0.02). Specifically, the association between animal protein intake and all-cause mortality was significant in male participants [Q4 versus Q1: 1.42 (1.20, 1.68), per 5 E% increment: 1.42 (1.13, 1.77)]; while the association was null in female participants [Q4 versus Q1: 1.01 (0.87, 1.17), per 5 E% increment: 1.04 (0.90, 1.21)]. There was no interaction between protein intake and sex for cause-specific mortality. The results were similar with the adjustment for the modified diet quality score without main protein food sources (data not shown). Last, the results were similar after excluding deaths cases within the first 2 years of follow-up (Supplemental Table 5).

### Meta-analysis results of the Rotterdam Study and previous prospective cohort studies

#### Literature search and characteristics of studies

In the initial search, we identified 12,152 potentially relevant unique citations. After screening and detailed full-text assessment, ten previously published articles were eligible for the systematic review [7–14, 16, 40]. Finally, ten previous studies were eligible for the meta-analysis [7–14, 16, 40], resulting in a total of eleven prospective studies including the Rotterdam study, with a total number of 350,452 participants and 64,306 deaths (Supplemental Figure 2). Furthermore, the number of participants (from 1100 to 131,342) and deaths (from 60 to 36,115) varied widely across these eleven studies. Median duration of follow-up ranged from 12.0 to 28.0 years. Among the eleven studies, most study populations were middle-aged at baseline (Table 6). Moreover, of the eleven studies, eight [8–11, 13, 14, 40] were conducted among North American and European populations (87% of total participants of this meta-analysis), in which the mean or median intake of total protein ranged from around 70 through 93 g/day, mainly from animal protein intake with a mean or median ranging from around 54 through 65 g/day. Three studies were conducted within Japanese populations, with a total of 82,171 participants [7, 12, 16]. Detailed characteristics and quality assessment of these studies have been summarized in Table 6 and Supplemental Table 6. Overall, all the eleven studies were medium to high quality.

**Table 6:** Characteristics of the prospective studies included in the systematic review and meta-analysis

Author, publication year	Study cohort/populations	Country	Baseline age (years, mean or range)	Female (%)	Follow-up (years)	Number of participants	Number of deaths			Level of adjustment	NOS^2^ score
							All deaths	CVD deaths	Cancer deaths		
Sauvaget et al. [7]	The Adult Health Study (AHS)	Japan	57	100	14	3731	NA	60	NA	++	7
Kelemen et al. [8]	The Iowa Women’s Health Study,	US	55–69	100	16.4	29,017	3978	739	1676	+++	8
Smit et al. [9]	The Puerto Rico Heart Health Program (PRHHP)	Puerto Rico	45–64	0	12	9777	NA	NA	167	++	8
Bates et al.^1^ [40]	The community-living population of mainland Britain	UK	76.7	50.2	14	1100	749	199	Na	+	6
Levine et al. [10]	NHANES III	US	64.8	55.4	13.1	6381	2553	1212	638	+++	8
Song et al. [11]	Nurses’ Health Study and Health Professional Follow-up study	US	49	64.7	27.0	131,342	36,115	8851	13,159	+++	9
Tharrey et al.^1^ [14]	The Adventist Health Study 2 (AHS-2)	US and Canada	> 25	NA	9.4	81,337	NA	2276	NA	+++	9
Kurihara et al. [12]	The National Integrated Project for Prospective Observation of Non-communicable Disease and Its Trends in the Age 1990 (NIPPONDATA90)	Japan	52.6	58.4	13.9	7744	1213	354	NA	+++	9
Virtanen et al. [13]	The Kuopio Ischaemic Heart Disease Risk Factor Study (KIHD)	Finland	52.7–53.7	0	22.31	2641	1225	618	347	+++	9
Budhathoki et al. [16]	Japan Public Health Center–based Prospective Cohort (JPHC) Study	Japan	55.7	54.5	18	70,696	12,381	3025	5055	+++	9
Chen et al. current study	The Rotterdam Study	Netherlands	63.5	60.8	13.0	7786	3589	877	896	+++	9

Level of adjustment: +, minimally adjusted (typically adjusted for age, sex, CVD confounders including BMJ but not for other nutritional factors); ++, adjusted for other macronutrients and/or other nutritional factors; +++, adjusted for subtypes of protein (e.g. animal and plant protein intake). All the studies adjusted for BMI

*CVD death* cardiovascular diseases death, *Newcastle*–*Ottawa Scale* score with a theoretical range from zero to nine with higher scores reflecting higher study design quality

^1^Inclusion only in the systematic review (and in sensitivity meta-analysis of dose–response), not in the main meta-analysis because of different format of estimates. The two studies only reported estimates of continuous variable of protein intake with the outcomes, did not report estimates of highest versus lowest category of protein intake

^2^NOS score, Newcastle–Ottawa Scale score with a theoretical range from zero to nine with higher scores reflecting higher study design quality

### Meta-analyzed associations for protein intake and all-cause and cause-specific mortality

#### Highest versus lowest meta-analysis

**Fig. 1:**
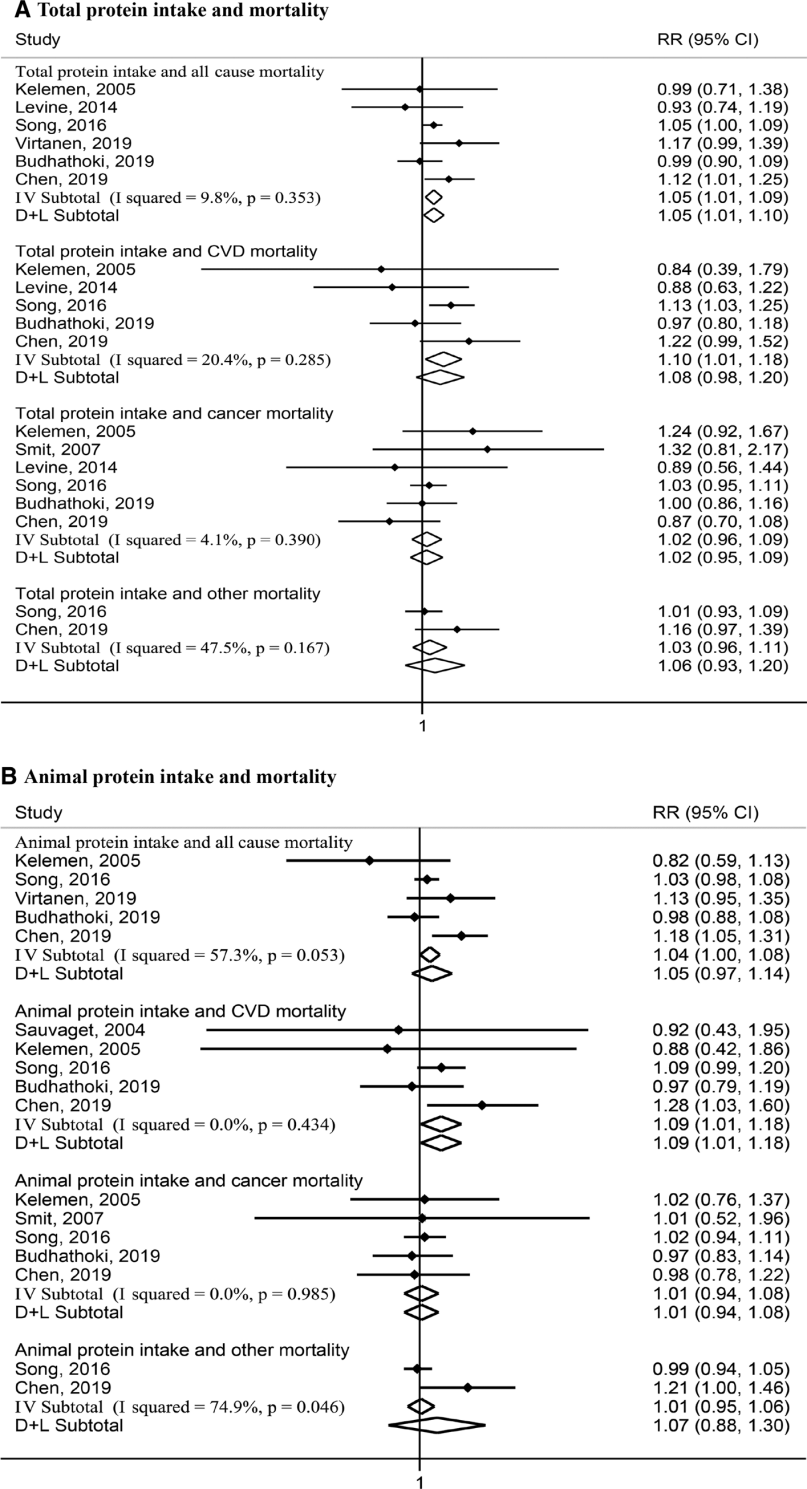

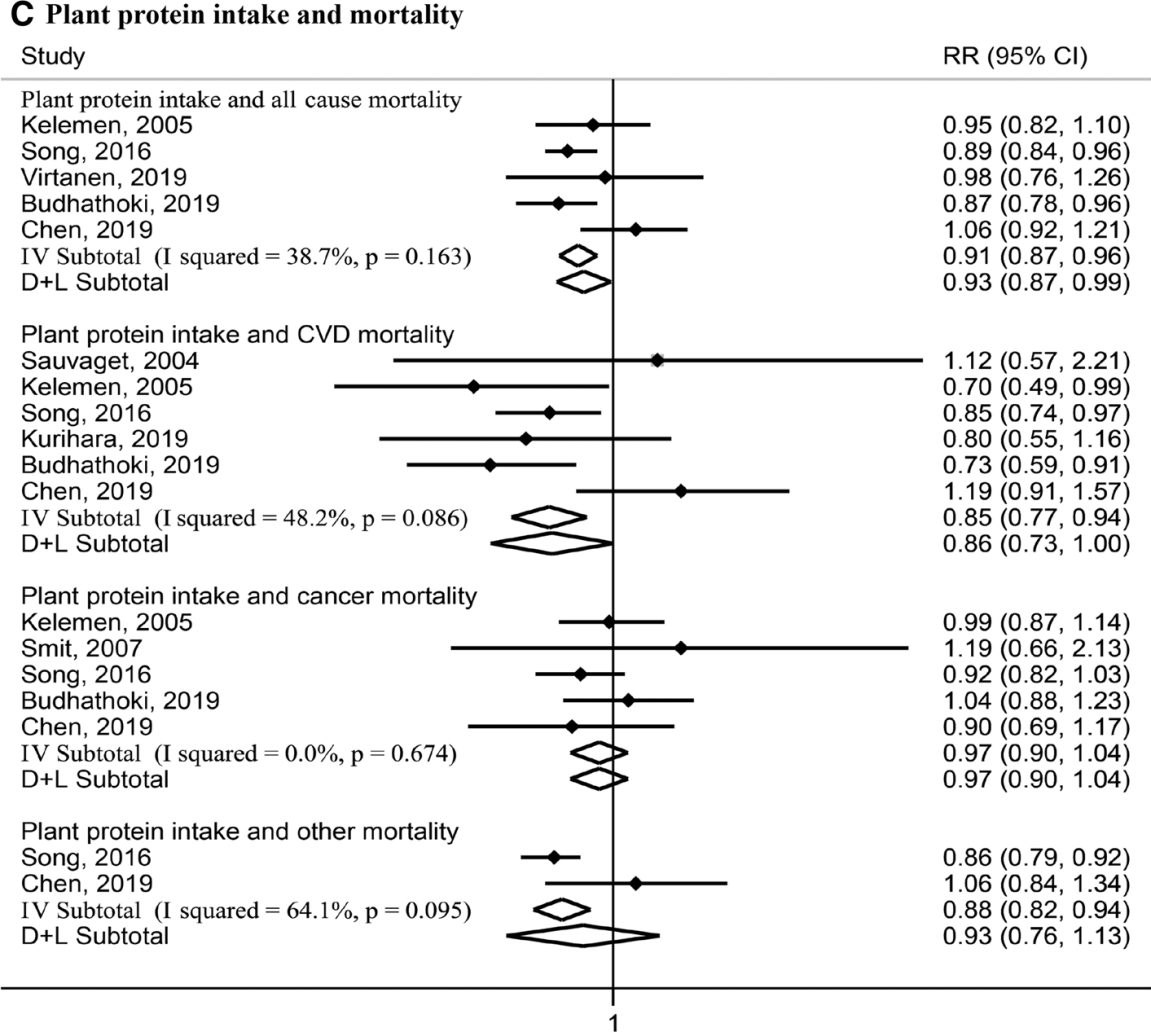
Relative risks (RRs) for the associations between protein intake (highest versus lowest categories) with all-cause and cause-specific mortality. Solid dots denote individual HRs, horizontal lines demote individual 95% CIs, open diamonds correspond to the pooled RRs including the 95% CIs, *P* values denote *P*_heterogeneity_ values, I–V Subtotal denotes fixed-effects analysis, and D + L Subtotal denotes random-effects analysis. *CVD mortality* cardiovascular mortality, *RR relative risk*, *CI* confidential interval. **a**, 59,841 all-cause deaths among 247,863 participants for total protein and all-cause mortality, 14,704 CVD deaths among 245,222 participants for total protein and CVD mortality, 21,591 cancer deaths among 25,499 participants for total protein and cancer mortality, 15,394 other deaths and 139,128 participants for total protein and other mortality. **b** 57,288 all-cause deaths among 241,482 participants for animal protein and all-cause mortality, 13,552 CVD deaths among 242,572 participants for animal protein and CVD mortality, 15,898 cancer deaths among 248,618 participants for animal protein and cancer mortality, 15,394 other deaths and 139,128 participants for animal protein and other mortality. **c** 57,288 all-cause deaths among 241,482 participants for plant protein and all-cause mortality, 13,906 CVD deaths among 250,316 participants for plant protein and CVD mortality, 28,953 cancer deaths among 248,618 participants for plant protein and cancer mortality, 15,394 other deaths and 139,128 participants for plant protein and other mortality

Nine studies [7–13, 16] including the Rotterdam Study presented associations of comparing the highest with the lowest categories of protein intake with mortality, and thus were summarized into the highest versus lowest meta-analysis. Figure 1 shows the results of the highest versus lowest meta-analysis. Of the nine studies, six examined associations [8, 10, 11, 13, 16] for total protein intake with all-cause mortality (59,841 all-cause deaths among 247,863 participants). Comparing the highest quantile of total protein intake with the lowest quantile, the pooled RR was 1.05, 95%CI (1.01, 1.10), I^2^ = 9.8%, *P*_heterogeneity_ = 0.35 for all-cause mortality. Five studies [8, 10, 11, 16] examined associations for total protein and CVD mortality (14,704 CVD deaths among 245,222 participants), with a pooled estimate of 1.08, 95%CI (0.98, 1.20), I^2^ = 20.4%, *P*_heterogeneity_ = 0.29. Six studies [8–11, 16] examined associations for total protein and cancer mortality; and two studies [11] on other mortality. For both these outcomes, pooled RRs were null (Fig. 1a).

For animal protein intake, five studies reported associations with all-cause mortality [8, 11, 13, 16], CVD mortality [7, 8, 11, 16], or cancer mortality [8, 9, 11, 16], and two studies [11] with other mortality (Fig. 1b). While null pooled associations were observed for all-cause, cancer, and other mortality, a significant pooled RR was observed for CVD mortality: 1.09, 95%CI (1.01, 1.18), I^2^ = 0.0%, *P*_heterogeneity_ = 0.43. For plant protein intake, similar studies were included with a pooled RR of 0.93, 95%CI (0.87, 0.99), I^2^ = 38.7%, *P*_heterogeneity_ = 0.16 for all-cause mortality, and 0.86 (0.73, 1.00), I^2^ = 48.2%, *P*_heterogeneity_ = 0.09 for CVD mortality. We observed null associations for plant protein and cancer mortality and other mortality (Fig. 1c).

#### Dose–response meta-analysis

**Fig. 2:**
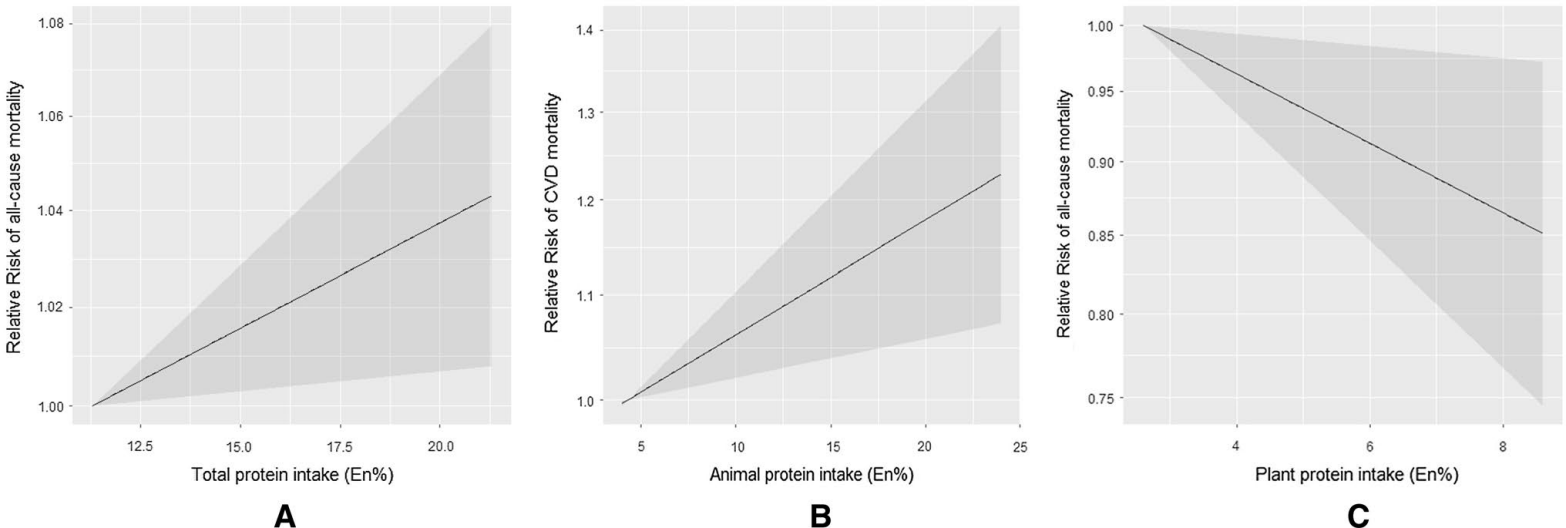
Combined dose–response associations between dietary protein intake with mortality (solid line) with 95% confidence intervals (shaded area). **a** The median total protein intake ranged from 11.3 E% through 25.0 E%. **b** The median animal protein intake ranged from 4.3 E% through 20.0 E%. **c** The median plant protein intake ranged from 2.6 E% through 8.4 E%

We performed dose–response meta-analysis based on six studies [10–13, 16] (Supplemental Table 7), from which sufficient data could be extracted to estimate dose–response estimates. In these studies, the median animal protein intake ranged from 4.3 E% through 20.0 E%, and plant protein from 2.6 E% through 8.4 E%. We found no evidence for non-linear associations (Wald test: *P* > 0.05). In line with the highest versus lowest meta-analysis, we observed a positive linear association between total protein intake and all-cause mortality (per 5 E% increment, 1.02 (1.004, 1.04), I^2^ = 37.9%, *P*_heterogeneity_ = 0.17), mainly driven by animal protein intake and CVD mortality [Per 5 E% increment, 1.05 (1.02, 1.09), I^2^ = 31.2%, *P*_heterogeneity_ = 0.23)] (Supplemental Table 7, Fig. 2a, b). Furthermore, we observed an inverse linear association between plant protein intake with all-cause mortality (per 5 E% increment, 0.87 (0.78, 0.98), I^2^ = 40.0%, *P*_heterogeneity_ = 0.17) (Supplemental Table 7, Fig. 2c). We observed no dose–response associations for the other examined associations (Supplemental Table 7).

#### Subgroup and sensitivity meta-analysis

We observed that several meta-analysis results were modified by geographical study location (Supplemental Table 8). For total protein and all-cause mortality and for animal protein and CVD mortality, positive associations were observed in North American and European populations, whereas null associations were observed in Japanese populations. For plant protein, inverse associations with all-cause and CVD mortality were only observed in North American and Japanese populations, but not in European populations (Supplemental Table 8). For the sensitivity analyses, as shown in Supplemental Table 9, the pooled associations were similar after excluding one study at each turn; and thus, were not driven by one individual study. Supplemental Table 10 shows the results of the second set of sensitivity analysis in which we included two additional studies in the dose–response meta-analysis that did not report associations for protein in E% but rather in g/day [14] or in SD [40]. After incorporating results from the study by Bates et al. [40] for total protein intake with all-cause mortality and CVD mortality, the pooled dose–response association between total protein intake and all-cause mortality was null, but with high heterogeneity (I^2^ = 87.8%, *P*_heterogeneity_ = 0.004) (Supplemental Table 10). Estimates for animal and plant protein were not available in this study. After incorporating results from the study by Tharrey et al. [14] for animal and plant protein and CVD mortality, the results remained similar [e.g. for animal protein and CVD mortality: per 5 E% increment, 1.08 (1.01, 1.16)] (Supplemental Table 10).

The appearance of funnel plots was symmetrical for all analyses, and Egger’s test results were not significant (Supplemental Figure 3), suggesting no publication bias.

## Discussion

### Main findings

In the Rotterdam Study, we observed that higher total protein intake was associated with higher all-cause mortality, which was mainly driven by higher animal protein intake and CVD mortality. Plant protein intake was not associated with all-cause or cause-specific mortality. A meta-analysis of eleven prospective cohort studies including the Rotterdam Study corroborated that higher total protein intake may increase risk of all-cause mortality, driven by a harmful association between animal protein and CVD mortality. Furthermore, our overall meta-analysis also indicated that higher plant protein may decrease all-cause mortality and CVD mortality. These overall meta-analysis results were modified by geographical study location. As we further observed that the harmful associations of total and animal protein were mainly among the North American and European populations, and the inverse associations of plant protein were mainly among the North American and Japanese populations.

### Interpretations of our findings

In contrast to reported beneficial short-term effects of dietary protein intake on weight management, and cardiovascular risk factors [2, 3, 41], we observed that a higher total protein intake was associated with higher all-cause mortality, which was mainly driven by a positive association between animal protein intake and CVD mortality.

Possible mechanisms and pathways for animal protein and CVD mortality may involve the amino acids of animal protein (e.g. branched-chain and aromatic amino acids) and accompanying components of animal protein from animal food sources (e.g. SFA from red and processed meat). Animal protein is relatively high in dietary branched-chain and aromatic amino acids, which may result in insulin resistance [42, 43] and overweight [43, 44], via mammalian target of rapamycin (mTOR) pathway [45]. These are strong risk factors for various cardiometabolic diseases, in turn increasing CVD mortality risk [42]. Furthermore, the association could be fueled or amplified by other components in animal-based foods, such as SFA and sodium from red and processed meat, which have both been linked to higher CVD risk [11, 46]. To investigate if the association of animal protein intake and CVD mortality would differ by more specific CVD causes, we further examined non-stroke CVD mortality and stroke mortality in the Rotterdam Study. We observed that the association of animal protein and CVD mortality was mainly driven by non-stroke CVD mortality, which is in line with previous studies which indicated a lack of association between animal protein intake with stroke [16, 47]. Moreover, we observed in subgroup analyses that these harmful associations were mainly observed in North American and European populations, not in Japanese populations. That could be partly explained by different levels and food sources of animal protein intake. In the North American [11] and European study populations [13], the major animal protein sources were red and processed meat; whereas in the Japanese study populations, population levels of animal protein intake were lower and the main animal protein source was fish [16]. Indeed, in the Rotterdam Study, we observed that the harmful association of animal protein intake and CVD mortality was mainly driven by protein from meat and dairy not by protein from fish and eggs. Similarly, Song et al. [11] also reported that the harmful association of mainly driven by protein from red and processed meat not by fish and eggs among American cohorts. In addition to different food sources, differences in cooking or preparation methods may also have contributed to the discrepancy of the associations between the Japanese, North American and European populations [48, 49]. For plant protein, we observed inverse associations between plant protein intake and all-cause and CVD mortality in the overall meta-analysis. Overall, the difference of associations for animal protein and plant protein might be explained by their different amino acid composition. Unlike animal protein, plant protein is generally low in branched-chain acids and aromatic amino acids [5, 50], thereby, resulting in decreased risks of CVD [43]. In subgroup analyses, we observed the inverse associations existed in North American and Japanese populations, but not in European populations. This may also be explained by different dietary plant protein sources among different populations. In the European populations, the main source was grains [42]. Among the North American populations in the studies by Song et al. and Malik et al., the main plant protein sources were legumes, whole grains and nuts [11, 51], and in the Japanese populations in the study by Budhathoki et al. [16], the main source was legumes. Indeed, we observed that in the Rotterdam Study protein from grains as the main contributor of plant protein, was not associated with all-cause and cause-specific mortality, yet, protein from legumes, nuts, vegetables, and fruits, was inversely associated with all-cause and cause-specific mortality. This may be explained by differences in other components in these food groups, such as fibers and unsaturated fat [52].

Overall, the evidences provided herein indicates the importance of specific protein sources for overall health, especially CVD health, and support a replacement of animal protein intake with plant protein intake. For example, in our meta-analysis, we observed that those in the highest quantile of animal protein intake, may have an averagely 9% higher CVD mortality risk than those in the lowest quantile. Based on reports of the individual studies, we estimated that those in the highest quantile had a median animal protein intake of approximately 75 g/day, and those in the lowest quantile around 38 g/day. This suggests that a decrease in animal protein intake from 75 g/day (e.g. corresponding to around 220 g red meat/day) to 42 g/day (e.g., around 100 g red meat/day), may attenuate risk of CVD mortality by around 9%, assuming other covariates remain stable. However, given that the studies in our meta-analysis were mainly performed among general middle-aged populations, our results and public health implications cannot be generalized to patient groups who may have other protein requirements and very old people. For example, for severely ill patients or very old people, high dietary protein intake may be beneficial in recovery or to prevent sarcopenia.

### Strengths and limitations

Our study has several strengths. First, the Rotterdam Study analysis was based on a prospective design and included comprehensive assessments of cause-specific deaths. Second, our meta-analysis is, to our knowledge, the first to summarize the associations of specific dietary protein intake with all-cause and cause-specific mortality, for which, we conducted not only highest versus lowest meta-analyses, but also dose–response meta-analyses. This can help to quantify the associations and test the shape of these possible associations. Third, the meta-analysis was based on several prospective cohort studies across various populations from different geographical locations. Moreover, the combined sample size was large, and the follow-up period was long, resulting in a substantial number of cases. Additionally, the cohort studies cohort studies in the meta-analysis were of medium to high quality, and their analyses included macronutrient substitution models as well as adjustments for other important confounding factors, such as total energy, physical activity, and BMI.

We also need to acknowledge several limitations. First, the Rotterdam Study and most studies in the meta-analysis measured dietary intake data based on self-reported FFQs, 24-h dietary recalls, or food records, for which measurements errors are unavoidable (e.g. dietary under-reporting). However, as these methods were expected to adequately rank subjects according to food and nutrient intake, we do not expect these measurement-errors to have largely affected associations. Second, in all studies except one, dietary intake data were measured only once at baseline, and changes in diet over time may affect associations. However, our results were generally consistent with results from the only study with repeated dietary measurements [11]. Third, in the Rotterdam Study analysis, a weak trend of an association between animal protein intake and other mortality might exist, but we could not further explore this due to limited numbers of cases for death from specific other causes. Fourth, we observed that the geographic study location modified the meta-analysis results. However, we could not further conduct subgroup analyses or meta-regression to explore other potential sources of the heterogeneity (e.g., age and sex). For example, we could not explore possible sex difference. Only two studies including the Rotterdam Study reported sex-stratified associations. The Rotterdam Study analysis observed that the association between animal protein intake and all-cause mortality, but not CVD mortality, differed by sex in the Rotterdam Study, with positive associations only in men. Only one other study examined sex differences for this association and observed null associations in both genders [11]. Fifth, we examined associations of protein from more specific food sources (e.g. protein from meat, fish, dairy, grains, and potatoes) with mortality in the Rotterdam Study, but the data for protein from more specific food sources and mortality to date are still limited, and we were not able to further meta-analyze evidence for protein from more specific food sources. Sixth, since all the studies included in the meta-analysis were conducted in general middle-aged and young-old populations (< 65 years) at baseline, our results may not be generalizable to populations with other protein requirements. Last, as a meta-analysis of observational studies, the results could be subject to residual or unmeasured confounding. Thus, the associations we report should be interpreted with caution.

In conclusion, our study provides evidence that higher total protein intake is associated with higher all-cause mortality, primarily driven by a positive association between animal protein intake and CVD mortality. In contrast, higher plant protein intake is associated with lower all-cause and CVD mortality. Food source and level of protein may play a substantial role as we observed harmful associations of total and animal protein mainly in North American and European populations and beneficial associations of plant protein mainly in North American and Japanese populations. Further studies in other populations with different amounts and food sources of protein intakes or with different protein requirements are needed to improve global dietary recommendations and to define optimal ranges and sources of protein intake for different populations.

## Electronic supplementary material

Below is the link to the electronic supplementary material.

Supplementary material 1 (DOCX 120 kb)

Supplementary material 2 (PPTX 447 kb)
