# Supplementary material for: Dietary protein intake and all-cause and cause-specific mortality: results from the Rotterdam Study and a meta-analysis of prospective cohort studies
Source: Eur J Epidemiol. 2020 Feb 19;35(5):411–29. doi: 10.1007/s10654-020-00607-6 (PMC7250948; doi:10.1007/s10654-020-00607-6)
Supplement: Supplementary file 1 — Supplementary material 1 (DOCX 120 kb) [file 10654_2020_607_MOESM1_ESM.docx]

**Dietary protein intake and all-cause and cause-specific mortality: results from the Rotterdam Study and a meta-analysis of prospective cohort studies**

**Supplemental Table 1 - Missing values in the Rotterdam Study (n=7,786)**

|  | **Physical activity** | **BMI** | **Education level** | **Smoking status** |
| --- | --- | --- | --- | --- |
| **RS-I n=4,309** | NA=1,515 (35.1%) | NA=29 (0.67%) | NA=23 (0.53%) | NA=28 (0.64%) |
| **RS-II n=1,249** | NA=6 (0.48%) | NA=4 (0.32%) | NA=14 (1.1%) | NA=5 (0.40%) |
| **RS-III n=2,228** | NA=195 (8.8%) | NA=62 (2.8%) | NA=7 (0.31%) | NA=6 (0.27%) |
| **Total n=7,786** | NA=1,716 (22.0%) | NA=95 (1.2%) | NA=44 (0.56%) | NA=39 (0.50%) |

In our main analyses, only four variables: physical activity, BMI, education level, and smoking status were with missing values.

Abbreviations: NA, not available, (numbers of participants with missing values); BMI, body mass index, RS, Rotterdam Study.

**Supplemental Table 2 –Detailed search terms and strategies**

| **Database** | **Search term** |
| --- | --- |
| **embase.com** | ('protein diet'/exp OR 'protein intake'/de OR 'plant protein'/de OR 'red meat'/exp OR 'dairy product'/exp OR 'nut'/exp OR 'soybean protein'/exp OR 'soybean milk'/exp OR 'protein restriction'/exp OR meat/exp OR (protein/de AND 'diet supplementation'/de) OR (((protein* OR nut OR nuts OR meat) NEAR/3 (intake OR diet* OR consum* OR nutrion OR food OR eating OR restrict* OR suppl* OR added OR rich OR enrich* OR meal*)) OR red-meat OR (milk NOT (breast-milk OR human-milk)) OR dairy OR cheese OR ((plant* OR animal OR soy) NEXT/1 protein*) OR yogurt OR yoghurt ):ab,ti) AND ('cardiovascular disease'/de OR 'heart failure'/de OR 'congestive heart failure'/de OR 'heart disease'/de OR 'coronary artery disease'/de OR 'ischemic heart disease'/exp OR 'cerebrovascular accident'/de OR 'atherosclerotic cardiovascular disease'/de OR 'brain ischemia'/exp OR 'mortality'/exp OR 'diabetes mellitus'/de OR 'non insulin dependent diabetes mellitus'/de OR 'cardiovascular risk'/de OR (((cardiovascular OR coronar*) NEAR/3 (disease* OR event*)) OR cvd OR cvds OR ((ischemi* OR ischaemi* OR fail* OR insufficien*) NEAR/3 (heart OR cardia*)) OR (cerebrovascular* NEAR/3 accident*) OR cva OR stroke* OR ((brain OR cerebral) NEAR/3 (ischemi* OR ischaemi*)) OR mortalit* OR (diabet* NOT ((type-1 OR type-I OR DM-1 OR DM-I OR t1d OR gestation* OR iddm) NOT (type-2 OR type-ii OR type-2a OR type-iia OR type-2b OR type-iib OR DM-2 OR DM-ii OR t2d ))) OR niddm OR t2d OR t2dm OR ((chd OR cvd OR cardiovascul*) NEAR/3 risk*)):ab,ti) NOT ([animals]/lim NOT [humans]/lim) NOT ([Conference Abstract]/lim OR [Letter]/lim OR [Note]/lim OR [Editorial]/lim) AND ('cohort analysis'/exp OR 'prospective study'/exp OR 'longitudinal study'/exp OR 'retrospective study'/exp OR 'follow up'/de OR 'case control study'/exp OR 'cross-sectional study'/exp OR 'clinical study'/exp OR 'meta analysis'/de OR 'clinical trial'/exp OR 'major clinical study'/de OR ((cross NEXT/1 section*) OR (case NEXT/1 control*) OR cohort* OR trial* OR ((clinical OR prospectiv* OR population* OR observation* OR retrospecti* OR intervention* ) NEAR/3 stud*) OR 'follow up' OR (meta NEXT/1 analy*) OR metaanaly* OR trial OR random*):ab,ti) |
| **Medline ovid** | (Diet, Protein-Restricted/ OR exp Diet, High-Protein/ OR exp Dietary Proteins/ OR exp Protein Deficiency/ OR exp Plant Proteins/ OR exp Dairy Products/ OR exp nuts/ OR exp Soy Foods/ OR exp meat/ OR (proteins/ AND Dietary Supplements/) OR (((protein* OR nut OR nuts OR meat) ADJ3 (intake OR diet* OR consum* OR nutrion OR food OR eating OR restrict* OR suppl* OR added OR rich OR enrich* OR meal*)) OR red-meat OR (milk NOT (breast-milk OR human-milk)) OR dairy OR cheese OR ((plant* OR animal OR soy) ADJ protein*) OR yogurt OR yoghurt ).ab,ti.) AND (Cardiovascular Diseases/ OR heart failure/ OR Heart Diseases/ OR Coronary Artery Disease/ OR exp Myocardial Ischemia/ OR stroke/ OR exp brain ischemia/ OR exp mortality/ OR exp Survival/ OR diabetes mellitus/ OR Diabetes Mellitus, Type 2/ OR (((cardiovascular OR coronar*) ADJ3 (disease* OR event*)) OR cvd OR cvds OR ((ischemi* OR ischaemi* OR fail* OR insufficien*) ADJ3 (heart OR cardia*)) OR (cerebrovascular* ADJ3 accident*) OR cva OR stroke* OR ((brain OR cerebral) ADJ3 (ischemi* OR ischaemi*)) OR mortalit* OR (diabet* NOT ((type-1 OR type-I OR DM-1 OR DM-I OR t1d OR gestation* OR iddm) NOT (type-2 OR type-ii OR type-2a OR type-iia OR type-2b OR type-iib OR DM-2 OR DM-ii OR t2d ))) OR niddm OR t2d OR t2dm OR ((chd OR cvd OR cardiovascul*) ADJ3 risk*)).ab,ti.) NOT (exp animals/ NOT humans/) NOT (letter OR news OR comment OR editorial OR congresses OR abstracts).pt. AND (exp Cohort Studies/ OR Case-Control Studies/ OR cross-sectional study/ OR Meta-Analysis / OR exp clinical trial/ OR ((cross ADJ section*) OR (case ADJ control*) OR cohort* OR trial* OR ((clinical OR prospectiv* OR population* OR observation* OR retrospecti* OR intervention* ) ADJ3 stud*) OR follow up OR (meta ADJ analy*) OR metaanaly* OR trial OR random*).ab,ti.) |
| **Cochrane CENTRAL** | ((((protein* OR nut OR nuts OR meat) NEAR/3 (intake OR diet* OR consum* OR nutrion OR food OR eating OR restrict* OR suppl* OR added OR rich OR enrich* OR meal*)) OR red-meat OR (milk NOT (breast-milk OR human-milk)) OR dairy OR cheese OR ((plant* OR animal OR soy) NEXT/1 protein*) OR yogurt OR yoghurt ):ab,ti) AND ((((cardiovascular OR coronar*) NEAR/3 (disease* OR event*)) OR cvd OR cvds OR ((ischemi* OR ischaemi* OR fail* OR insufficien*) NEAR/3 (heart OR cardia*)) OR (cerebrovascular* NEAR/3 accident*) OR cva OR stroke* OR ((brain OR cerebral) NEAR/3 (ischemi* OR ischaemi*)) OR mortalit* OR (diabet* NOT ((type-1 OR type-I OR DM-1 OR DM-I OR t1d OR gestation* OR iddm) NOT (type-2 OR type-ii OR type-2a OR type-iia OR type-2b OR type-iib OR DM-2 OR DM-ii OR t2d ))) OR niddm OR t2d OR t2dm OR ((chd OR cvd OR cardiovascul*) NEAR/3 risk*)):ab,ti) |

**Supplemental Table 3 – Characteristics of population of the Rotterdam Study across the quartiles of total, animal, and plant protein (n=7,786)**

|  | **Total protein** | | | |  | **Animal protein** | | | |  | **Plant protein** | | | |
| --- | --- | --- | --- | --- | --- | --- | --- | --- | --- | --- | --- | --- | --- | --- |
| **Mean ± SD** | **85.8 ± 25.1 g/day, 16.4% ± 2.3 E%** | | | |  | **53.6 ± 19.0 g/day, 10.3% ± 2.5% E%** | | | |  | **30.3 ± 8.9 g/day, 6.2% ± 1.5% E%** | | | |
|  | **Q1** | **Q2** | **Q3** | **Q4** |  | **Q1** | **Q2** | **Q3** | **Q4** |  | **Q1** | **Q2** | **Q3** | **Q4** |
|  | **n=1,947** | **n=1,946** | **n=1,946** | **n=1,947** |  | **n=1,947** | **n=1,946** | **n=1,946** | **n=1,947** |  | **n=1,947** | **n=1,946** | **n=1,946** | **n=1,947** |
|  | **≤14.4%** | **14.4%>,**  **≤16.2%** | **16.2%>, ≤18.1%** | **>18.1%** |  | **≤8.4%** | **8.4%>, ≤10.2%** | **10.2%>,**  **≤12.2%** | **>12.2%** |  | **≤5.2%** | **5.2%>, ≤5.9%** | **5.9%>, ≤6.7%** | **>6.7%** |
| **Age (Year)** | 63.9  (9.4) | 63.1  (8.8) | 63.5  (8.3) | 64.2  (8.2) |  | 62.1  (9.1) | 63.7  (8.8) | 64.1  (8.5) | 64.9  (8.2)* |  | 66.2  (8.8) | 65.1  (8.7) | 62.9  (8.3) | 60.6  (7.9)* |
| **Sex (%)** |  |  |  |  |  |  |  |  |  |  |  |  |  |  |
| -Female | 12.8 | 14.4 | 15.7 | 18.4* |  | 12.9 | 13.9 | 16.0 | 17.9* |  | 13.9 | 13.7 | 15.7 | 15.6* |
| -Male | 12.7 | 10.6 | 9.3 | 6.6* |  | 12.1 | 11.0 | 9.0 | 7.1* |  | 11.1 | 9.4 | 9.3 | 9.4 |
| **BMI (kg/m^2^)** | 25.8  (3.6) | 26.3  (3.7) | 26.6  (3.8) | 27.5*  (4.3) |  | 25.8  (3.7) | 26.3  (3.7) | 26.7  (3.9) | 27.4*  (4.2)* |  | 26.4  (3.7) | 26.5  (3.8) | 26.8  (4.0) | 26.6  (4.1) |
| **Smoking Status (%)** |  |  |  |  |  |  |  |  |  |  |  |  |  |  |
| -Never | 7.2 | 8.3 | 8.5 | 9.3 |  | 7.8 | 8.1 | 8.5 | 8.9 |  | 6.8 | 8.6 | 8.9 | 9.1 |
| -Ever | 11.0 | 10.7 | 10.5 | 10.1 |  | 11.4 | 10.9 | 10.3 | 9.8 |  | 9.6 | 10.8 | 10.7 | 11.2 |
| -Current | 6.7 | 5.9 | 5.8 | 5.4* |  | 5.7 | 5.9 | 6.0 | 6.2* |  | 8.5 | 5.4 | 5.3 | 4.7* |
| **Education level (%)** |  |  |  |  |  |  |  |  |  |  |  |  |  |  |
| -Primary | 3.6 | 3.6 | 4.0 | 4.1 |  | 3.1 | 3.7 | 4.0 | 4.5 |  | 4.6 | 4.3 | 3.2 | 3.1 |
| -Low | 10.0 | 9.9 | 10.3 | 10.9 |  | 9.5 | 10.3 | 10.3 | 10.9 |  | 10.8 | 10.3 | 10.4 | 9.6 |
| -Intermediate | 7.2 | 6.9 | 6.6 | 6.6 |  | 7.1 | 6.8 | 7.0 | 6.4 |  | 6.6 | 6.9 | 7.2 | 6.5 |
| -High | 4.1 | 4.5 | 4.0 | 3.2 |  | 5.2 | 4.0 | 3.6 | 3.1* |  | 2.9 | 3.3 | 4.0 | 5.7* |
| **Physical activity**  **(MET-hours/week)** |  |  |  |  |  |  |  |  |  |  |  |  |  |  |
| -RS-I and II | 74.3  (47.8, 105.6) | 79.0  (56.2, 112.1) | 81.5  (55.9, 113.4) | 83.9  (59.8, 117.5)* |  | 76.7  (49.5,  108.7) | 78.8  (56.2, 112.0) | 80.7  (55.7, 113.6) | 83.0 (58.1, 116.0)* |  | 75.9  (47.6, 105.5) | 80.1  (57.4, 113.8) | 81.8  (56.4, 112.7) | 84.8 (61.6, 121.0)* |
| -RS-III | 41.0  (17.1,  84.3) | 49.2  (21.0,  81.5) | 42.2  (15.4, 82.9) | 39.4 (15.0, 56.4) |  | 42.8  (18.0,  84.1) | 51.5  (21.0,  82.7) | 38.5  (13.6,  83.3) | 38.0 (15.0, 72.4) |  | 38.0  (16.0, 72.4) | 36.5  (15.3, 73.0) | 45.1  (18.1,  86.6) | 48.0 (18.7, 87.8)* |
| **Dietary intake** |  |  |  |  |  |  |  |  |  |  |  |  |  |  |
| **Protein from meat^1^ (E%)** | 3.1  (1.4) | 3.8  (1.6) | 4.6  (1.8) | 5.6  (2.6)* |  | 2.8  (1.3) | 3.9  (1.5) | 4.6  (1.8) | 5.8  (2.6)* |  | 4.8  (2.4) | 4.5  (2.0) | 4.3  (2.0) | 3.7  (2.0)* |
| **Protein from dairy^2^ (E%)** | 3.0  (1.4) | 3.8  (1.6) | 4.5  (2.0) | 6.0  (2.8)* |  | 2.8  (1.3) | 3.8  (1.5) | 4.6  (1.9) | 6.1  (2.8)* |  | 4.7  (2.5) | 4.5  (2.2) | 4.3  (2.2 | 3.8  (2.1)* |
| **Protein from fish (E%)** | 0.5  (0.6) | 0.7  (0.8) | 0.8  (0.9) | 1.1*  (1.2) |  | 0.6  (0.6) | 0.7  (0.8) | 0.8  (0.9) | 1.0*  (1.2) |  | 0.7  (1.0) | 0.8  (0.9) | 0.8  (0.8) | 0.8  (0.9) |
| **Protein from eggs (E%)** | 0.3  (0.3) | 0.4  (0.3) | 0.4  (0.3) | 0.4  (0.3) |  | 0.3  (0.3) | 0.3  (0.3) | 0.3  (0.3) | 0.3  (0.3) |  | 0.4  (0.3) | 0.4  (0.3) | 0.4  (0.3) | 0.3  (0.3) |
| **Protein from grains (E%)** | 2.9  (1.1) | 3.1  (1.1) | 3.1  (1.1) | 3.1  (1.2) |  | 3.3  (1.2) | 3.1  (1.0) | 3.0  (1.1) | 2.8*  (1.1) |  | 2.2  (0.8) | 2.8  (0.8) | 3.3  (0.9) | 3.9*  (1.2) |
| **Protein from potatoes (E%)** | 0.4  (0.3) | 0.4  (0.3) | 0.5  (0.3) | 0.5  (0.3) |  | 0.4  (0.3) | 0.5  (0.3) | 0.5  (0.3) | 0.5  (0.3) |  | 0.4  (0.2) | 0.5  (0.3) | 0.5  (0.3) | 0.4  (0.3) |
| **Protein from legumes, nuts, fruits, vegetables (E%)** | 1.8  (1.4) | 1.9  (1.4) | 1.9  (1.5) | 2.0*  (1.7) |  | 2.4  (1.8) | 1.8  (1.3) | 1.7  (1.3) | 1.7*  (1.4) |  | 1.2  (1.0) | 1.5  (1.1) | 1.9  (1.2) | 3.0*  (1.9) |
| **Total fat (E%)** | 35.3  (7.3) | 35.8  (6.4) | 35.7  (6.1) | 34.5  (6.2) |  | 34.3  (7.3) | 35.4  (6.1) | 36.1  (6.2) | 35.6  (6.5)* |  | 38.1  (7.1) | 36.0  (6.0) | 34.7  (5.7) | 32.3  (6.0)* |
| **SFA (E%)** | 13.3  (3.4) | 13.6  (3.3) | 13.8  (3.3) | 13.5  (3.3) |  | 12.4  (3.3) | 13.5  (3.0) | 14.1  (3.2) | 14.2  (3.4)* |  | 15.5  (3.5) | 14.1  (3.0) | 13.2  (2.7) | 11.5  (2.6)* |
| **MUFA (E%)** | 11.9.  (3.2) | 11.9.  (2.7) | 11.9  (2.5) | 11.5  (2.5) |  | 11.5  (3.2) | 11.9  (2.7) | 11.9  (2.7) | 11.9  (2.7) |  | 12.8  (3.2) | 11.9  (2.7) | 11.5  (2.5) | 11.0  (2.7)* |
| **PUFA (E%)** | 7.2  (2.7) | 7.2  (2.5) | 7.0  (2.3) | 6.5  (2.5) |  | 7.4  (2.7) | 7.2  (2.5) | 7.0  (2.5) | 6.5  (2.5)* |  | 6.8  (2.7) | 7.0  (2.5) | 7.0  (2.3) | 7.2  (2.3)* |
| **TSF (E%)** | 0.72  (0.52,  1.10) | 0.72  (0.52,  1.10) | 0.74  (0.54, 1.06) | 0.72 (0.54, 0.97) |  | 0.61  (0.45,  0.92) | 0.72  (0.52,  1.10) | 0.79  (0.56,  1.10) | 0.77 (0.59, 1.06) |  | 0.92  (0.65, 1.33) | 0.81 (0.59, 1.13) | 0.70 (0.52, 0.97) | 0.56 (0.43, 0.74)* |
| **Diet quality** | 6.3  (2.0) | 6.7  (1.9) | 6.9  (1.8) | 7.2  (1.8)* |  | 6.8  (2.0) | 6.6  (1.9) | 6.7  (1.8) | 6.9  (1.8) |  | 5.7  (1.7) | 6.5  (1.7) | 7.0  (1.8) | 7.7  (1.7)* |
| **Fiber (gram)** | 20.8  (15.4,  28.9) | 20.6  (15.7,  28.6) | 19.6  (15.2, 26.4) | 17.7  (14.3, 22.6)* |  | 24.6  (18.1,  33.9) | 20.4  (15.8,  27.5) | 18.5  (14.8,  239) | 16.6 (13.6, 20.9)* |  | 15.2  (12.2, 19.5) | 18.1 (14.7, 23.3) | 20.8 (16.6, 27.4) | 26.2 (19.6, 35.2)* |

Variables expressed as mean (SD), median (25th percentile–75th percentile), or percentage.

P-trend was assessed were tested with linear regression (continuous variables) or with chi-square test (categorical variables). * indicates P < 0.05 for trend across quartiles.

^1^ Red and processed meat comprised 80% of meat products.

^2^ Milk and cheese comprised 75% of dairy products.

Abbreviations: MET, metabolic equivalent; E%, energy percent; SFA, saturated fat acids, MUFA, monounsaturated fat acids, PUFA, polyunsaturated fat acids, TSF, trans saturated fat acids.

**Supplemental Table 4 - Associations of protein with all-cause and cause-specific mortality (n=7,786, comparison isocaloric substitution for fat)**

| **Dietary protein** | **HR (95% CI) per 5 E% increment** | | **Quartile 1** | **Quartile 2** | **Quartile 3** | | **Quartile 4** | | | **P trend** |
| --- | --- | --- | --- | --- | --- | --- | --- | --- | --- | --- |
| n= 7,786 |  | | n= 1,906 | n=1,906 | n=1,905 | | n=1,906 | | |  |
|  | **Total protein** | | | | | | | | | |
| **Median intake (E%)** | 16.2 | 13.3 | | 15.3 | 17.0 | | | 19.7 | |  |
| **All-cause mortality** |  |  | |  |  | | |  | |  |
| Number of deaths | n=3,589 | n=1,176 | | n=986 | n=813 | | | n=614 | |  |
| Multivariate model | 1.08 (1.01, 1.15) | 1 (Reference) | | 1.04 (0.94, 1.15) | 1.01 (0.91, 1.12) | | | 1.10 (0.98, 1.22) | | 0.04 |
| **Cardiovascular mortality** |  |  | |  |  | | |  | |  |
| Number of deaths | n=877 | n=169 | | n=216 | n=219 | | | n=273 | |  |
| Multivariate model | 1.14 (0.99, 1.30) | 1 (Reference) | | 0.99 (0.81, 1.20) | 0.98 (0.79, 1.20) | | | 1.20 (0.97, 1.49) | | 0.07 |
| **- Non-stroke CVD mortality** |  |  | |  |  | | |  | |  |
| Number of deaths | n=594 | n=109 | | n=145 | n=150 | | | n=190 | |  |
| Multivariate model | 1.17 (1.004, 1.38) | 1 (Reference) | | 0.95 (0.74, 1.22) | 1.00 (0.78, 1.28) | | | 1.20 (0.92, 1.55) | | 0.13 |
| **- Stroke mortality** |  |  | |  |  | | |  | |  |
| Number of deaths | n=283 | n=92 | | n=80 | n=62 | | | n=49 | |  |
| Multivariate model | 1.07 (0.84, 1.35) | 1 (Reference) | | 1.05 (0.75, 1.49) | 0.93 (0.64, 1.34) | | | 1.22 (0.84, 1.79) | | 0.34 |
| **Cancer mortality** |  |  | |  |  | | |  | |  |
| **Number of deaths** | n=896 | n=305 | | n=227 | n=204 | | | n=160 | |  |
| Multivariate model | 0.93 (0.81, 1.06) | 1 (Reference) | | 0.96 (0.79, 1.16) | 0.90 (0.73, 1.08) | | | 0.87 (0.70, 1.08) | | 0.16 |
| **Other mortality** |  |  | |  |  | | |  | |  |
| Number of deaths | n=1,289 | n=441 | | n=392 | n=261 | | | n=195 | |  |
| Multivariate model | 1.07 (0.96, 1.20) | 1 (Reference) | | 1.11 (0.94, 1.31) | 1.06 (0.89, 1.26) | | | 1.16 (0.96, 1.39) | | 0.18 |
|  | **Animal protein** | | | | | | | | | |
| **Median intake (E%)** | 10.2 | | 7.2 | 9.3 | | 11.1 | | | 13.9 |  |
| **All-cause mortality** |  | |  |  | |  | | |  |  |
| Number of deaths | n=3,589 | | n=1,176 | n=986 | | n=813 | | | n=614 |  |
| Multivariate model | 1.16 (1.02, 1.33) | | 1 (Reference) | 1.05 (0.95, 1.17) | | 1.07 (0.97, 1.19) | | | 1.17 (1.04, 1.30) | 0.001 |
| **Cardiovascular mortality** |  | |  |  | |  | | |  |  |
| Number of deaths | n=877 | | n=169 | n=216 | | n=219 | | | n=273 |  |
| Multivariate model | 1.13 (0.99, 1.30) | | 1 (Reference) | 1.06 (0.86, 1.30) | | 0.99 (0.80, 1.22) | | | 1.24 (0.99, 1.55) | 0.05 |
| **Non-stroke CVD mortality** |  | |  |  | |  | | |  |  |
| Number of deaths | n=594 | | n=109 | n=145 | | n=150 | | | n=190 |  |
| Multivariate model | 1.19 (1.002, 1.39) | | 1 (Reference) | 1.11 (0.86, 1.43) | | 1.05 (0.81, 1.38) | | | 1.34 (1.01, 1.75) | 0.04 |
| **Stroke mortality** |  | |  |  | |  | | |  |  |
| Number of deaths | n=283 | | n=92 | n=80 | | n=62 | | | n=49 |  |
| Multivariate model | 1.05 (0.83, 1.34) | | 1 (Reference) | 1.00 (0.70, 1.42) | | 0.88 (0.61, 1.28) | | | 1.09 (0.90, 2.03) | 0.69 |
| **Cancer mortality** |  | |  |  | |  | | |  |  |
| Number of deaths | n=896 | | n=305 | n=227 | | n=204 | | | n=160 |  |
| Multivariate model | 0.93 (0.81, 1.07) | | 1 (Reference) | 1.11 (0.90, 1.34) | | 0.96 (0.77, 1.19) | | | 0.98 (0.78, 1.23) | 0.56 |
| **Other mortality** |  | |  |  | |  | | |  |  |
| Number of deaths | n=1,289 | | n=441 | n=392 | | n=261 | | | n=195 |  |
| Multivariate model | 1.09 (0.98, 1.22) | | 1 (Reference) | 1.04 (0.88, 1.24) | | 1.13 (0.95, 1.35) | | | 1.24 (1.02, 1.49) | 0.02 |
|  | **Plant protein** | | | | | | | | | |
| **Median intake (E%)** | 5.9 | | 4.7 | 5.5 | | 6.2 | | | 7.3 |  |
| **All-cause mortality** |  | |  |  | |  | | |  |  |
| Number of deaths | n=3,589 | | n=1,176 | n=986 | | n=813 | | | n=614 |  |
| Multivariate model | 1.04 (0.85, 1.27) | | 1 (Reference) | 0.93 (0.85, 1.02) | | 0.92 (0.83, 1.01) | | | 1.00 (0.88, 1.13) | 0.75 |
| **Cardiovascular mortality** |  | |  |  | |  | | |  |  |
| Number of deaths | n=877 | | n=169 | n=216 | | n=219 | | | n=273 |  |
| Multivariate model | 1.22 (0.82, 1.81) | | 1 (Reference) | 0.98 (0.82, 1.18) | | 1.08 (0.88, 1.32) | | | 1.19 (0.93, 1.53) | 0.14 |
| **Non-stroke CVD mortality** |  | |  |  | |  | | |  |  |
| Number of deaths | n=594 | | n=109 | n=145 | | n=150 | | | n=190 |  |
| Multivariate model | 1.17 (0.79, 1.73) | | 1 (Reference) | 0.92 (0.74, 1.15) | | 1.07 (0.85, 1.35) | | | 1.12 (0.84, 1.48) | 0.31 |
| **Stroke mortality** |  | |  |  | |  | | |  |  |
| Number of deaths | n=283 | | n=92 | n=80 | | n=62 | | | n=49 |  |
| Multivariate model | 1.39 (0.80, 2.44) | | 1 (Reference) | 1.06 (0.77, 1.45) | | 1.07 (0.76, 1.52) | | | 1.36 (0.90, 2.03) | 0.16 |
| **Cancer mortality** |  | |  |  | |  | | |  |  |
| Number of deaths | n=896 | | n=305 | n=227 | | n=204 | | | n=160 |  |
| Multivariate model | 0.83 (0.59, 1.16) | | 1 (Reference) | 0.82 (0.68, 0.98) | | 0.84 (0.70, 1.03) | | | 0.89 (0.71, 1.11) | 0.27 |
| **Other mortality** |  | |  |  | |  | | |  |  |
| Number of deaths | n=1,289 | | n=441 | n=392 | | n=261 | | | n=195 |  |
| Multivariate model | 0.77 (0.58, 1.02) | | 1 (Reference) | 1.00 (0.86, 1.15) | | 0.81 (0.69, 0.96) | | | 0.92 (0.76, 1.12) | 0.18 |

Effect estimates are hazard ratios (HRs) and 95%-confidence intervals (95%CIs) derived from Cox proportional hazards regression models with adjustment for carbohydrate (E%), total energy, alcohol (E%), fiber, age, sex, RS-cohorts (RS-I, -II, and -III), education level (primary, lower, intermediate, and high), smoking status (never, ever, current), physical activity (z-score of metabolic equivalent of task-hours/week), diet quality score, BMI. Animal protein and plant protein were mutually adjusted. Estimates are based on pooled results of imputed data. Abbreviations: BMI, body mass index.

**Supplemental Table 5 - Associations of protein and all-cause and cause-specific mortality after excluding death cases within the first 2 years of follow-up in the Rotterdam study (n=7,623, comparison isocaloric substitution for carbohydrate)**

| **Dietary protein** | HR (95% CI) per 5 E% increment | **Quartile 1** | **Quartile 2** | **Quartile 3** | **Quartile 4** | **P trend** |
| --- | --- | --- | --- | --- | --- | --- |
|  | n= 7,623 | n= 1,906 | n=1,906 | n=1,905 | n=1,906 |  |
|  | **Total protein** | | | | | |
| **Median intake (E%)** | 16.2 | 13.3 | 15.3 | 17.0 | 19.7 |  |
| **All-cause mortality** |  |  |  |  |  |  |
| Number of deaths | n=3,427 | n=825 | n=811 | n=850 | n=941 |  |
| Multivariate model | 1.09 (1.02, 1.17) | 1 (Reference) | 1.06 (0.96, 1.19) | 1.04 (0.93, 1.16) | 1.15 (1.02, 1.28) | 0.04 |
| **Cardiovascular mortality** |  |  |  |  |  |  |
| Number of deaths | n=806 | n=200 | n=175 | n=186 | n=245 |  |
| Multivariate model | 1.16 (1.00, 1.35) | 1 (Reference) | 0.99 (0.79, 1.23) | 1.02 (0.82, 1.28) | 1.26 (1.00, 1.60) | 0.05 |
| **- Non-stroke CVD mortality** |  |  |  |  |  |  |
| Number of deaths | n=539 | n=131 | n=112 | n=127 | n=169 |  |
| Multivariate model | 1.23 (1.04, 1.48) | 1 (Reference) | 0.93 (0.72, 1.22) | 1.05 (0.80, 1.38) | 1.34 (1.00, 1.77) | 0.04 |
| **- Stroke mortality** |  |  |  |  |  |  |
| Number of deaths | n=267 | n=69 | n=63 | n=59 | n=76 |  |
| Multivariate model | 1.01 (0.76, 1.32) | 1 (Reference) | 1.12 (0.76, 1.63) | 0.95 (0.63, 1.43) | 1.09 (0.70, 1.70) | 0.91 |
| **Cancer mortality** |  |  |  |  |  |  |
| **Number of deaths** | n=836 | n=220 | n=204 | n=207 | n=205 |  |
| Multivariate model | 0.95 (0.82, 1.12) | 1 (Reference) | 0.92 (0.75, 1.14) | 0.90 (0.73, 1.12) | 0.90 (0.70, 1.15) | 0.38 |
| **Other mortality** |  |  |  |  |  |  |
| Number of deaths | n=1,258 | n=303 | n=304 | n=312 | n=339 |  |
| Multivariate model | 1.09 (0.97, 1.23) | 1 (Reference) | 1.14 (0.95, 1.35) | 1.04 (0.87, 1.26) | 1.17 (0.96, 1.43) | 0.22 |
|  | **Animal protein** | | | | | |
| **Median intake (E%)** | 10.2 | 7.2 | 9.3 | 11.1 | 13.9 |  |
| **All-cause mortality** |  |  |  |  |  |  |
| Number of deaths | n=3,427 | n=656 | n=839 | n=929 | n=1,003 |  |
| Multivariate model | 1.09 (1.02, 1.17) | 1 (Reference) | 1.08 (0.97, 1.21) | 1.11 (0.99, 1.23) | 1.21 (1.07, 1.36) | 0.01 |
| **Cardiovascular mortality** |  |  |  |  |  |  |
| Number of deaths | n=806 | n=154 | n=201 | n=200 | n=251 |  |
| Multivariate model | 1.16 (1.00, 1.35) | 1 (Reference) | 1.09 (0.88, 1.38) | 1.03 (0.82, 1.30) | 1.32 (1.04, 1.70) | 0.04 |
| **Non-stroke CVD mortality** |  |  |  |  |  |  |
| Number of deaths | n=539 | n=97 | n=134 | n=133 | n=175 |  |
| Multivariate model | 1.23 (1.03, 1.48) | 1 (Reference) | 1.13 (0.86, 1.49) | 1.12 (0.86, 1.43) | 1.38 (1.04, 1.82) | 0.04 |
| **Stroke mortality** |  |  |  |  |  |  |
| Number of deaths | n=267 | n=57 | n=67 | n=67 | n=76 |  |
| Multivariate model | 1.00 (0.77, 1.32) | 1 (Reference) | 1.03 (0.69, 1.54) | 0.98 (0.65, 1.48) | 1.03 (0.66, 1.62) | 0.17 |
| **Cancer mortality** |  |  |  |  |  |  |
| Number of deaths | n=836 | n=168 | n=226 | n=216 | n=226 |  |
| Multivariate model | 0.95 (0.82, 1.12) | 1 (Reference) | 1.01 (0.80, 1.26) | 0.93 (0.74, 1.17) | 0.98 (0.76, 1.26) | 0.41 |
| **Other mortality** |  |  |  |  |  |  |
| Number of deaths | n=1,258 | n=233 | n=294 | n=356 | n=375 |  |
| Multivariate model | 1.09 (0.97, 1.23) | 1 (Reference) | 1.05 (0.88, 1.27) | 1.11 (0.91, 1.34) | 1.25 (1.00, 1.52) | 0.06 |
|  | **Plant protein** | | | | | |
| **Median intake (E%)** | 5.9 | 4.7 | 5.5 | 6.2 | 7.3 |  |
| **All-cause mortality** |  |  |  |  |  |  |
| Number of deaths | n=3,427 | n=1,110 | n=943 | n=787 | n=587 |  |
| Multivariate model | 1.08 (0.87, 1.36) | 1 (Reference) | 0.96 (0.87, 1.05) | 0.95 (0.84, 1.06) | 1.07 (0.93, 1.23) | 0.68 |
| **Cardiovascular mortality** |  |  |  |  |  |  |
| Number of deaths | n=806 | n=251 | n=218 | n=200 | n=137 |  |
| Multivariate model | 1.35 (0.85, 2.14) | 1 (Reference) | 1.04 (0.85, 1.28) | 1.07 (0.84, 1.36) | 1.26 (0.93, 1.70) | 0.43 |
| **Non-stroke CVD mortality** |  |  |  |  |  |  |
| Number of deaths | n=539 | n=165 | n=143 | n=141 | n=90 |  |
| Multivariate model | 1.31 (0.75, 2.29) | 1 (Reference) | 1.05 (0.82, 1.34) | 1.09 (0.82, 1.45) | 1.25 (0.87, 1.80) | 0.56 |
| **Stroke mortality** |  |  |  |  |  |  |
| Number of deaths | n=267 | n=86 | n=75 | n=59 | n=47 |  |
| Multivariate model | 1.45 (0.64, 3.25) | 1 (Reference) | 1.04 (0.72, 1.51) | 1.04 (0.68, 1.60) | 1.30 (0.75, 2.23) | 0.35 |
| **Cancer mortality** |  |  |  |  |  |  |
| Number of deaths | n=836 | n=285 | n=214 | n=190 | n=147 |  |
| Multivariate model | 0.79 (0.49, 1.30) | 1 (Reference) | 0.84 (0.68, 1.03) | 0.85 (0.67, 1.07) | 0.90 (0.66, 1.20) | 0.14 |
| **Other mortality** |  |  |  |  |  |  |
| Number of deaths | n=1,258 | n=433 | n=384 | n=250 | n=191 |  |
| Multivariate model | 1.09 (0.63, 1.40) | 1 (Reference) | 1.07 (0.91, 1.26) | 0.87 (0.71, 1.05) | 1.08 (0.84, 1.39) | 0.89 |

Effect estimates are hazard ratios (HRs) and 95%-confidence intervals (95%CIs) derived from Cox proportional hazards regression models with adjustment for SFA (E%), MUFA (E%), PUFA (E%), TSF (E%), total energy, alcohol (E%), fiber, age, sex, RS-cohorts (RS-I, -II, and -III), education level(primary, lower, intermediate, and high), smoking status (never, ever, current), physical activity (z-score of metabolic equivalent of task-hours/week), diet quality score, BMI. Animal protein and plant protein were mutually adjusted. Estimates are based on pooled results of imputed data. Abbreviations: SFA, saturated fat acids, MUFA, monounsaturated fat acids, PUFA, polyunsaturated fat acids, TSF, trans fat acids; BMI, body mass index.

**Supplemental Table 6 – Characteristics of studies in the systematic review and the meta-analysis**

| **The first author, publication year** | **Study name** | **Country** | | **Baseline age (year)** | **Female**  **(%)** | **Follow-up (years)** | **Number of participants** | | **Number of deaths** | | | **Level of adjustment** | **NOS^2^ score** |
| --- | --- | --- | --- | --- | --- | --- | --- | --- | --- | --- | --- | --- | --- |
|  |  |  |  |  |  |  |  |  | All | CVD | Cancer |  |  |
| Sauvaget et al., 2004 (1) | The Adult Health Study (AHS) | Japan | | 35-89 | 100 | 14 | 3,731 | | NA | 60 | NA | **++** | 7 |
| Kelemen et al., 2005 (2) | The Iowa Women’s Health Study, | US | | 55-69 | 100 | 16.4 | 29,017 | | 3,978 | 739 | 1,676 | **+++** | 8 |
| Smit et al,2007 (3) | The Puerto Rico Heart Health Program (PRHHP) | Puerto Rico | | 45-64 | 0 | 12 | 9,777 | | NA | NA | 167 | **++** | 8 |
| Bates et al, 2010^1^ (4) | The community-living population of mainland Britain | UK | | 76·7 | 50.2 | 14 | 1,100 | | 749 | 199 | Na | + | 6 |
| Levine et al., 2014 (5) | NHANES III | US | | 64.8 | 55.4 | 13.1 | 6,381 | | 1,851 | 879 | 463 | **+++** | 8 |
| Song et al., 2016 (6) | Nurses’ Health Study  And  Health Professional Follow-up study | US | | 49 | 64.7 | 27.0 | 131,342 | | 36,115 | 13,159 | 14,105 | **+++** | 9 |
| Tharrey et al., 2018^1^ (7) | The Adventist Health Study 2 (AHS-2) | US and Canada | | >25 | NA | 9.4 | 81,337 | | NA | 2,276 | NA | **+++** | 9 |
| Kurihara et al, 2019 (8) | The National Integrated Project for Prospective  Observation of Non-communicable Disease and Its Trends in the Aged 1990 (NIPPON DATA90) | Japan | | 52.6 | 58.4 | 13.9 | 7,744 | | 1,213 | 354 | Na | **+++** | 9 |
| Virtanen et al, 2019 (9) | The Kuopio Ischaemic Heart Disease Risk Factor Study (KIHD) | Finland | | 52.7-53.7 | 0 | 22.31 | 2,641 | | 1,225 | 618 | 347 | **+++** | 9 |
| Budhathoki et al, 2019 (10) | Japan Public Health Center–based Prospective Cohort (JPHC) Study | Japan | | 45-74 | 54.5% | 18 | 70,696 | | 12,381 | 3,025 | 5,055 | +++ | 9 |
| Chen et al., 2019 | The Rotterdam Study | Netherlands | | 63.5 | 60.8 | 13.0 | 7,786 | | 3,589 | 846 | 835 | **+++** | 9 |
| Level of adjustment: +, minimally adjusted (typically adjusted for age, sex, CVD confounders but not for the other nutritional factors); ++, adjusted for fat/vitamin/supplements use; +++, adjusted for protein types (e.g. adjusted for animal and plant protein intake).  ^1^indicates inclusion only in the systematic review and in a sensitivity meta-analysis not in the main sensitivity analysis.  ^2^ NOS score, Newcastle–Ottawa Scale, score with a theoretical range from zero to nine with higher scores reflecting higher study design quality | | | | | | | | | | | | | |
| **The first author**  **and publication year** | **Exposure assessment** | | **Exposure categorization** | | | | | **Outcome assessment** | | | **Variables in fully adjusted models** | | |
| Sauvaget et al., 2004 (1) | A diet questionnaire that included items on vitamin supplements, type of work, and body measurements. Diaries were provided and completed by the AHS participants equally throughout the week and the year. Also, a trained nurse provided the participants with instructions for completing the diary and with a measuring spoon for weighing foods. The subjects chose a day to record their usual meals and wrote down the names of the foods along with the amount consumed at each meal. A dietician then checked and coded the diaries and contacted the subjects if entries needed clarification. Nutrient consumption was estimated from the Japan Food Composition Tables. | | Exposure was categorized in tertiles (T) with the lowest tertile being used as a reference category (T1)  RR was reported T3 vs. T1.  (^a^TP, Mean intake: 92 g vs 48g)  (AP, Mean: 54 g vs 18g)  (PP, Mean: 46 g vs 24g) | | | | | Vital status was ascertained by linkage to the nationwide family registration system of Japan. Cause of death followed the International Classification of Diseases codes (9th and 10th revisions) | | | Age, sex, radiation dose, city, BMI, alcohol, medical history and diabetes, fruit and vegetable intake, fat intake, blood cholesterol level, total energy intake, body weight (For animal protein, adjusted all above, for TP, PP, ending with medical history) | | |
| Kelemen et al.,2005 (2) | Baseline diet was assessed by a validated semiquantitative food frequency questionnaire described by Rimm et al. For each food, a common unit or portion size was specified, and participants were asked how often, on average, they had consumed that amount of the item over the past year. The nine responses ranged from “never or less than once per month” to “six or more times per day.” The questionnaire also asked about vitamin and mineral supplement intake. Daily intakes of nutrients were calculated by summing across all food items the product of the frequency of consumption of the specified unit of each food by the nutrient content of that unit of food. The validity and reliability of the food frequency questionnaire have been documented in this cohort | | Exposure was categorized in quintiles (Q) with the lowest quintile being used as a reference category (Q1).  RR was reported as Q5 vs Q1  (TP, median 22.0 vs 14.1 en%)  (AP, 17.5 vs 8.9 en%)  (pp, 6.1 vs 3.7 en%) | | | | | CHD mortality, cancer mortality, total mortality, Incident cases of cancer were ascertained through the State Health Registry of Iowa, part of the Surveillance, Epidemiology, and End Results Program. | | | Age, total energy, SFA, PUFA, MUFA, TSF, fiber, dietary cholesterol, dietary methionine, alcohol, smoking, activity level, BMI, history of hypertension, postmenopausal hormone use, vitamin, vitamin E supplement use, education, and family history of cancer, AP and PP mutually adjusted | | |
| Smit et al.,2007 (3) | Dietary intake was assessed using a 24-h recall at baseline. Food models and standard-sized utensils were used to obtain a quantitative assessment from participants during a  24-h dietary recall. Intake of energy and macronutrients was calculated using the United States Department of Agriculture Handbook #8 food composition tables or other more direct sources of nutrients analysis for foods special to Puerto Rico. | | Exposure was categorized in quartile (Q) with the lowest quartile being used as the reference category (Q1).  (TP, range: ≥104g, vs. ≤61g)  (AP, range: ≥41, vs. ≤13g)  (PP, range: ≥32g, vs. ≤16g,)  Mean: TP, 85g/day, AP, 59.5g/day, TP, 25.5g/day | | | | | Prostate cancer mortality was assessed throughout the active phase of the study and using using passive follow-up by matching participants in the PRHHP with the Puerto Rico Cancer Registry and Puerto Rico Vital Statistics Registry. Validity of matched cases was assessed  by obtaining copies of the death certificate and using the Puerto Rico Cancer Registry | | | Age, sex, BMI, living, physical activity, smoking, energy intake, BMI, smoking status, alcohol intake, prevalence of diabetes, hypertension, hypercholesterolemia, family history of heart diseases, use of aspirin, antihypertensive medication, oral antidiabetic medication, insulin resistance, hypocholesterolemic medication, energy from fat, energy from carbohydrate, energy from fat acids, fiber, glycemic index. | | |
| Bates et al, 2010^1^ (4) | A 4-d weighed dietary record was also obtained by the interviewer | | Continuous exposure (gram, per SD) | | | | | All mortality and vascular mortality were assessed the National Register of Births and Deaths | | | age, sex, BMI, physical activity, alcohol consumption, receipt of welfare beneﬁt, cigarette smoking, energy intake, folate, vitamin B12, vitamin B6 intakes and plasma creatinine | | |
| Levine et al., 2014 (5) | Nutrient intake data are based on reports of food and beverage intake during a 24 hr period. Data were collected via an automated, microcomputer-based coding system, with information on over 80 nutrients. | | The percent of calorie intake from protein was used to categorize subjects into a high protein group (20% or more of calories from proteins), a moderate protein group (10–19% of calories from proteins), and a low protein group (less than 10% of calories from proteins).  (percentage of total energy)  (TP: >20% vs. <10 %)  (AP: continuous)  (TP: continuous) | | | | | Mortality follow-up was available for all NHANES III participants through linkage with the National Death Index | | | Age, sex, race/ethnicity, education, waist circumference, smoking, chronic conditions  (diabetes, cancer, MI), trying to lose in last year weight, diet changed in last year, reported intake representative of typical diet, total calories | | |
| Song et al., 2016 (6) | Dietary intake was assessed by the food frequency questionnaires (FFQs). Participants were asked how often, on average, they consumed a standardized portion size of each food during the previous year. The mean daily nutrient intake was calculated by multiplying the consumption frequency of each food Item by its nutrient content and then summing across all foods. Animal and plant protein intake was expressed as a percentage of total energy consumption. | | Exposure was categorized in quintiles (Q) with the lowest quintile being used as a reference category (Q1)  (percentage of total energy, median)  TP  AP: Q5 vs. Q1 20% vs. 8.9%;  PP: Q5 vs. Q1 6.6% vs. 2.6% | | | | | Deaths were identified from state statistics records, the National Death Index, next of kin, and the postal system. Cause of death was identified from death certificates or review of medical records by physicians. | | | Cox proportional hazards model with age as the time scale was stratified by sex and calendar time and adjustedfor total caloric intake and percentage of energy from saturated fat, polyunsaturated fat, monounsaturated fat,and trans-fat (all continuous), multivitamin use (yes or no), smoking status (never, past, or current [1-14, and15 cigarettes/d]), pack-years of smoking (in women, 15, 16-25, 26-45, and46; in men, <10, 11-24, 25-44, and45), BMI (<23·0, 23·0-24·9, 25·0-26·9, 27·0-29·9, 30·0-34·9, and35), physical activity (quintiles), alcohol consumption, history of hypertension diagnosis (yes or no), glycemic index (in quintiles), and intake of whole grains, total fiber, fruits,  and vegetables (all in quintiles). Mutual adjustment was conducted for animal protein and plant protein analysis | | |
| Tharrey et al., 2018^1^ (7) | Dietary habits were assessed using food frequency questionnaire (FFQ). The questionnaire was validated against six 24-h recalls. | | Each 18gram increase of animal protein or plant protein | | | | | Deaths were identified by biennial follow-up of participants and linkage with the National Death Index. The underlying cause of death was coded using the International Statistical Classification of Diseases and Related Health Problems, 10th Revision (ICD-10). CVD deaths were identified as those starting with the letter I. | | | Age, sex, race, energy intake, BMI, physical activity, smoking status, alcohol intake, marital status, type of diet in vegetarian spectrum, PUFA, MUFA, SFA, sodium and vitamins. | | |
| Kurihara et al, 2019 (8) | A dietary survey was carried out employing the weighing record method for three consecutive days in each household. Trained dietitians visited participants, and they were asked to weigh and record all foods and beverages that any of the household members consumed during the survey period. Dietitians visited participants’ homes at least once a day and confirmed the records during the survey. | | The intake of animal protein, vegetable protein, and fat were expressed as % energy. Sodium and potassium were expressed in mg, whereas other nutrients  were calculated as g/1000 kcal. The participants were divided into four categories according to the quartile of vegetable protein intake: vegetable protein intake ≤ 6.6%, 6.7% –7.2%, 7.3%–7.8%, and ≥ 7.9%. (percentage of total energy) | | | | | Causes of death were identified every five years by searching the National Vital Statistics database. The underlying causes of death identified by the National Vital Statistics were coded according to the 9th International Classification of Disease (ICD-9) for deaths up to the end of 1994 and the 10th International Classification of Disease (ICD-10) for deaths from 1995 onwards. | | | sex, age, BMI, animal protein intake, animal fat intake, vegetable fat intake, sodium, potassium, total dietary fiber, cigarette smoking category and alcohol intake category | | |
| Virtanen et al, 2019 (9) | The consumption of foods at baseline was assessed with an instructed food record of 4 d, 1 of which was a weekend day, by using household measures. | | Intakes of protein sources and energy-adjusted proteins were expressed as g/d in the analyses. Cox proportional hazards regression models were used to estimate HRs in exposure quartiles, with the lowest category as the reference.  (percentage of total energy, median)  TP: Q4 vs. Q1: 18.8% vs. 12.9%  AP: Q4 vs. Q1: 14.1% vs. 8.2%  PP: Q4 vs. Q1: 7.0% vs. 4.3% | | | | | Deaths were ascertained by a computer linkage to the national Causes of Death Register with the use of the Finnish personal  identification code (social security number). All deaths were coded according to the International Classification of Diseases (ICD), 10th revision, codes. All disease deaths that occurred from the study entry to 31 December, 2014, were included. Deaths due to accidents or suicides (ICD codes S00–T98) were not included. | | | Age, examination year, and energy intake (kilocalories per day), income (euros per year); education years; marital status (married/unmarried); leisure-time physical activity (kilocalories per day); pack-years of smoking (packs smoked per day × years smoked); alcohol intake (grams per week); BMI (kg/m2); and diagnosis of type 2 diabetes, cardiovascular disease, cancer, or hypertension or use of cardiac, hypercholesterolemia, hypertension, or diabetes medications (yes/no), intakes of fiber (grams per day) and  saturated, monounsaturated, polyunsaturated, and trans fatty acids (all grams per day) | | |
| Budhathoki et al, 2019 (10) | A semiquantitative food frequency questionnaire was used to assess usual intake of 138 food and beverage items during the previous year | | (percentage of total energy, median)  TP: Q5 vs. Q1: 17.6% vs. 11.3%  AP: Q5 vs. Q1: 11.2% vs. 4.3%  PP: Q5 vs. Q1: 8.4% vs. 5.0% | | | | | Residential and vital statuses of cohort participants during  follow-up was determined annually through the residential  registry. Causes of death, coded according to the International  Statistical Classification of Diseases and Related Health  Problems, Tenth Revision, were obtained from death certificates; all-cause mortality and deaths due to cancer (codes C00-C99), CVD (codes I00-I99), heart disease (codes I20-I52), and cerebrovascular disease (codes I60-I69). | | | 2 models: the first adjusted for age, sex, and percentage of energy from saturated,  monounsaturated, polyunsaturated, and other fats, whereas the second further adjusted for body mass index, smoking status, alcohol intake, total physical activity, coffee consumption,  green tea consumption, and total calorie intake while leaving  out the percentage of energy from carbohydrates. Mutual  adjustment for animal protein and plant protein in the respective analyses was performed | | |
| Chen et al., 2019 | FFQ with 170 food items was used at baseline of RS-I, and II. And FFQ with 389 food items was used at baseline of RS-III | | (percentage of total energy, median)  TP: Q4 vs. Q1: 19.7% vs. 13.3%  AP: Q4 vs. Q1: 13.9 % vs. 7.2%  PP: Q4 vs. Q1: 7.3 % vs. 4.6% | | | | | General practitioners report events of interest by means of a computerized system or notify new events annually. Trained research assistants subsequently collected information from medical records at the general practitioners’ offices, hospitals and nursing homes. Two research physicians independently identified the events according to the International Classification of Diseases, Tenth revision (ICD-10). Afterwards, a senior physician reviewed all coded events. Information on vital status of the participants was obtained from the clinical follow-up data collection described above and from municipal records. Cause-specific mortality was recoded according to the ICD-10 codes (CVD cause: F01, I05-99 (non-stroke CVD cause: F01, I05-51, 70-99 , stroke cause: I60-69); cancer cause: C01-97). Coded information on all-cause mortality was available until May 2018 and coded information on cause specific mortality was available until January 2014. | | | Age, sex, RS-cohorts, diet quality score, SFA, MUFA, PUFA, TSF, alcohol, fiber, total energy, physical activity, smoking, education, BMI | | |
| TP: total protein, AP: animal protein; PP: plant protein, Level of adjustment: +=minimally adjusted (typically adjusted for age, sex, CVD confounders but not for other nutritional factors); ++=adjusted for other macronutrients and/or other nutritional factors; +++=adjusted for subtypes of protein (e.g. animal and plant protein intake); NOS score, Newcastle–Ottawa Scale, score with a theoretical range from zero to nine with higher scores reflecting higher study design quality. ^a^ Result for total protein intake reported by Sauvaget et al. was not included in meta-analysis due to without adjustment for important confounders, such as total energy and fat. | | | | | | | | | | | | | |

**Supplemental Table 7 – Dose-response meta-analysis**

|  | **Number of studies** | **Number of participants** | **Number of deaths** | **RR (95% CI)** | **I^2^** | **P_heterogeneity_** |
| --- | --- | --- | --- | --- | --- | --- |
| **Total protein** |  |  |  |  |  |  |
| All-cause mortality | 5 (5, 6, 9) | 218,846 | 55,863 | 1.02 (1.004, 1.04) | 37.9% | 0.17 |
| CVD mortality | 4 (5, 6, 10) | 216,205 | 13,965 | 1.04 (0.997, 1.09) | 37.4% | 0.19 |
| Cancer mortality | 4 (5, 6, 10) | 216,205 | 19,748 | 1.00 (0.95, 1.05) | 11.6% | 0.33 |
| **Animal protein** |  |  |  |  |  |  |
| All-cause mortality | 4 (6, 9, 10) | 212,465 | 53,310 | 1.05 (0.99, 1.12) | 70.1% | 0.02 |
| CVD mortality | 3 (6, 10) | 209,824 | 12,753 | 1.05 (1.02, 1.09) | 31.2% | 0.23 |
| Cancer mortality | 3 (6, 10) | 209,824 | 19,110 | 1.01 (0.98, 1.04) | 0.0% | 0.76 |
| **Plant protein** |  |  |  |  |  |  |
| All-cause mortality | 4 (6, 9, 10) | 212,465 | 53,310 | 0.87 (0.78. 0.98) | 40.0% | 0.17 |
| CVD mortality | 4 (6, 8, 10) | 217,568 | 13,107 | 0.77 (0.52, 1.16) | 73.2% | 0.01 |
| Cancer mortality | 3 (6, 10) | 209,824 | 19,110 | 0.88 (0.71, 1.09) | 58.1% | 0.09 |

Risk ratios (RRs) and 95%-confidence intervals (95%CIs) reflect the difference in the risks per 5 En% increment of dietary protein intake based on a linear dose-response meta-analysis. When sufficient studies (n≥5) contributed to a dose-response meta-analysis, non-linearity of dose-response association was explored using restricted cubic splines with three knots (10%, 50%, and 90%) for the amount of dietary protein intake. No evidence for non-linear associations was observed (Wald test: p>0.05).

**Supplemental Table 8 - Subgroup meta-analysis by geographic study location**

|  | **Total protein** | | | | **Animal protein** | | | | **Plant protein** | | | | |
| --- | --- | --- | --- | --- | --- | --- | --- | --- | --- | --- | --- | --- | --- |
|  | **Number**  **of studies** | **RR (95% CI)** | **I^2^** | **P_heterogeneity_** | **Number**  **of studies** | **RR (95% CI)** | **I^2^** | **P_heterogeneity_** | **Number**  **of studies** | **RR (95% CI)** | **I^2^** | **P_heterogeneity_** |  |
| **All-cause mortality** |  |  |  |  |  |  |  |  |  |  |  |  |  |
| US | 3 | 1.05 (1.002, 1.09) | 0.0 | 0.58 | 2 | 0.97 (0.81, 1.18) | 46.0 | 0.17 | 3 | 0.90 (0.85, 0.96) | 0.0 | 0.43 |  |
| Europe | 2 | 1.13 (1.04, 1.24) | 0.0 | 0.67 | 2 | 1.17 (1.06, 1.28) | 0.0 | 0.68 | 2 | 1.04 (0.92, 1.17) | 0.0 | 0.59 |  |
| Asia | 1 | 0.99 (0.90,1.09) | - | - | 1 | 0.98 (0.88, 1.09) | - | - | 1 | 0.87 (0.78, 0.97) | - | - |  |
| **CVD mortality** |  |  |  |  |  |  |  |  |  |  |  |  |  |
| US | 3 | 1.06 (0.90, 1.25) | 20.8 | 0.28 | 2 | 1.09 (0.99, 1.19) | 0.0 | 0.58 | 3 | 0.77 (0.64, 0.92) | 0.0 | 0.49 |  |
| Europe | 1 | 1.22 (0.99 1.51) | - | - | 1 | 1.28 (1.03, 1.60) | - | - | 1 | 1.19 (0.91, 1.56) | - | - |  |
| Asia | 1 | 0.97 (0.80, 1.18) | - | - | 2 | 0.97 (0.79, 1.18) | 0.0 | 0.90 | 2 | 0.83 (0.73, 0.94) | 0.0 | 0.31 |  |
| **Cancer mortality** |  |  |  |  |  |  |  |  |  |  |  |  |  |
| US | 4 | 1.04 (0.97, 1.12) | 0.0 | 0.44 | 3 | 1.02 (0.94, 1.10) | 0.0 | 1.0 | 3 | 0.95 (0.87, 1.04) | 0.0 | 0.54 |  |
| Europe | 1 | 1.00 (0.86, 1.16) | - | - | 1 | 0.98 (0.78, 1.23) | - | - | 1 | 1.04 (0.88, 1.23) | - | - |  |
| Asia | 1 | 0.87 (0.70, 1.08) | - | - | 1 | 0.97 (0.83, 1.14) | - | - | 1 | 0.90 (0.69, 1.17) | - | - |  |

RR (95% CI), I^2^ and P_heterogeneity_ values were obtained from a highest versus lowest meta-analysis with random effects

**Supplemental Table 9 – Sensitivity analysis for meta-analysis by excluding each one study one at a time^1^**

|  | **RR (95% CI)** | **I^2^** | **P_heterogeneity_** |
| --- | --- | --- | --- |
| **Pooled association of total protein and all-cause mortality, after excluding:** |  |  |  |
| - Kelemen et al, 2005 (2) | 1.05 (1.00, 1.11) | 26.2% | 0.73 |
| - Levine et al, 2014 (5) | 1.06 (1.01, 1.10) | 11.3% | 0.31 |
| - Song et al, 2016 (6) | 1.05 (0.97, 1.14) | 27.8% | 0.97 |
| - Virtanen et al, 2019 (9) | 1.05 (1.01,1.08) | 0% | 0.20 |
| - Budhathoki et al, 2019 (10) | 1.06 (1.02, 1.10) | 0% | 0.19 |
| - Chen et al, 2019 | 1.04 (1.00, 1.08) | 0% | 0.21 |
| **Pooled association of total protein and CVD mortality, after excluding** |  |  |  |
| - Kelemen et al, 2005 (2) | 1.08 (0.96, 1.21) | 34.1% | 0.49 |
| - Levine et al, 2014 (5) | 1.11 (1.01, 1.21) | 7.4% | 0.18 |
| - Song et al, 2016 (6) | 1.03 (0.88, 1.21) | 23.1% | 0.29 |
| - Budhathoki et al, 2019 (10) | 1.12 (1.01,1.23) | 7.3% | 0.18 |
| - Chen et al, 2019 | 1.05 (0.93, 1.18) | 23.2% | 0.29 |
| **Pooled association of total protein and cancer mortality, after excluding** |  |  |  |
| - Kelemen et al, 2005 (2) | 1.01 (0.95, 1.08) | 0% | 0.19 |
| - Smit et al, 2007 (3) | 1.01 (0.95, 1.09) | 3.5% | 0.30 |
| - Levine et al, 2014 (5) | 1.02 (0.94, 1.11) | 18.2% | 0.57 |
| - Song et al, 2016 (6) | 1.01 (0.88, 1.15) | 20.6% | 0.67 |
| - Budhathoki et al, 2019 (10) | 1.02 (0.91,1.14) | 22.1% | 0.78 |
| - Chen et al, 2019 | 1.04 (0.97, 1.11) | 0% | 0.13 |
| **Pooled associations of animal protein and all-cause mortality, after excluding** |  |  |  |
| - Kelemen et al, 2005 (2) | 1.06 (0.98, 1.15) | 58.6% | 0.06 |
| - Song et al, 2016 (6) | 1.05 (0.93, 1.20) | 65.8% | 0.03 |
| - Virtanen et al, 2019 (9) | 1.04 (0.95,1.14) | 64.7% | 0.04 |
| - Budhathoki et al, 2019 (10) | 1.07 (0.97, 1.19) | 61.3% | 0.05 |
| - Chen et al, 2019 | 1.02 (0.96, 1.08) | 20.3% | 0.28 |
| **Pooled associations of animal protein and CVD mortality, after excluding** |  |  |  |
| - Sauvaget et al, 2004 (1) | 1.09 (0.99, 1.21) | 16.8% | 0.66 |
| - Kelemen et al, 2005 (2) | 1.09 (0.99, 1.21) | 13.9% | 0.57 |
| - Song et al, 2016 (6) | 1.08 (0.90, 1.30) | 21.1% | 0.97 |
| - Budhathoki et al, 2019 (10) | 1.11 (1.02, 1.21) | 0% | 0.23 |
| - Chen et al, 2019 | 1.06 (0.98, 1.16) | 0% | 0.12 |
| **Pooled associations of animal protein and cancer mortality, after excluding** |  |  |  |
| - Kelemen et al, 2005 (2) | 1.01 (0.94, 1.08) | 0% | 0.93 |
| - Smit et al, 2007 (3) | 1.01 (0.94, 1.08) | 0% | 0.99 |
| - Song et al, 2016 (6) | 0.98 (0.87, 1.10) | 0% | 0.60 |
| - Budhathoki et al, 2019 (10) | 1.02 (0.94, 1.09) | 0% | 0.61 |
| - Chen et al, 2019 | 1.01 (0.94, 1.08) | 0% | 0.80 |
| **Pooled associations of plant protein and all-cause mortality after excluding** |  |  |  |
| - Kelemen et al. 2005 (2) | 0.93 (0.85, 1.01) | 51.9% | 0.10 |
| - Song et al, 2016 (6) | 0.95 (0.86, 1.05) | 42.4% | 0.16 |
| - Virtanen et al, 2019 (9) | 0.93 (0.86,1.00) | 51.9% | 0.10 |
| - Budhathoki et al, 2019 (10) | 0.95 (0.87, 1.04) | 44.5% | 0.14 |
| - Chen et al, 2019 | 0.90 (0.85, 0.94) | 0% | 0.69 |
| **Pooled associations of plant protein and CVD mortality, after excluding** |  |  |  |
| - Sauvaget et al, 2004 (1) | 0.84 (0.71, 1.00) | 55.5% | 0.06 |
| - Kelemen et al, 2005 (2) | 0.88 (0.74, 1.05) | 52.4% | 0.08 |
| - Song et al, 2016 (6) | 0.86(0.68, 1.09) | 58.6% | 0.05 |
| - Kurihara et al, 2019 (8) | 0.87 (0.72, 1.04) | 58.1% | 0.05 |
| - Budhathoki et al, 2019 (10) | 0.90 (0.75,1.07) | 45.4% | 0.12 |
| - Chen et al, 2019 | 0.81 (0.73, 0.90) | 0% | 0.57 |
| **Pooled associations of plant protein and cancer mortality, after excluding** |  |  |  |
| - Kelemen et al, 2005 (2) | 0.95 (0.87, 1.04) | 0% | 0.66 |
| - Smit et al, 2007 (3) | 0.96 (0.89, 1.04) | 0% | 0.48 |
| - Song et al, 2016 (6) | 1.00 (0.91, 1.10) | 0% | 0.28 |
| - Budhathoki et al, 2019 (10) | 0.95 (0.87, 1.03) | 0% | 0.33 |
| - Chen et al, 2019 | 0.97 (0.90, 1.05) | 0% | 0.59 |

Effect estimates are Risk ratios (RRs) and 95% confidence intervals (95%CIs) derived from random-effect highest versus lowest meta-analysis. ^1^This sensitivity analysis was not conducted for other mortality, because there were only two studies this outcome.

**Supplemental Table 10 - Sensitivity analysis of dose-response meta-analysis**

|  | **RR (95% CI)** | **I^2^** | **P_heterogeneity_** |
| --- | --- | --- | --- |
| **Total protein and all-cause mortality** |  |  |  |
| The main dose-response meta-analysis results (Per 5 E%) | 1.02 (1,004, 1,04) | 37.9% | 0.17 |
| The result from the study by Bates et al (4) (Per 5 E%) | 0.86 (0.77, 0.97) | - | - |
| Pooled results (Per 5 E%) | 0.94 (0.80, 1.12) | 87.8% | 0.004 |
| **Total protein and CVD mortality** |  |  |  |
| The main dose-response meta-analysis results (Per 5 E%) | 1.04 (0,997, 1.09) | 37.4% | 0.19 |
| The result from the study by Bates et al (4) (Per 5 E%) | 0,79 (0.67, 0.94) | - | - |
| Pooled results (Per 5 E%) | 0.91 (0.70, 1.20) | 89.4% | 0.002 |
| **Animal protein and CVD mortality** |  |  |  |
| The main dose-response meta-analysis results (Per 5 E%) | 1.05 (1.02, 1.09) | 31.2% | 0.23 |
| The result from the study by Tharrey et al (7) (Per 5 E%) | 1.12 (1.05, 1.19) | - | - |
| Pooled results (Per 5 E%) | 1.08 (1.01, 1.16) | 68.6% | 0.07 |
| **Plant protein and CVD mortality** |  |  |  |
| The main dose-response meta-analysis results (Per 5 E%) | 0.77 (0.52, 1.16) | 73.2% | 0.01 |
| The result from the study by Tharrey (7) (Per 5 E%) | 0.95 (0.89, 1.05) | - | - |
| Pooled results (Per 5 E%) | 0.94 (0.87, 1.01) | 2.3% | 0.31 |
| Effect estimates are Risk ratios (RRs) and 95%-confidence intervals (95%CIs) derived from random-effect meta-analysis. | | | |

**References**

1. Sauvaget C, Nagano J, Hayashi M, Yamada M. Animal protein, animal fat, and cholesterol intakes and risk of cerebral infarction mortality in the adult health study. Stroke. 2004;35(7):1531-7.

2. Kelemen LE, Kushi LH, Jacobs Jr DR, Cerhan JR. Associations of dietary protein with disease and mortality in a prospective study of postmenopausal women. American journal of epidemiology. 2005;161(3):239-49.

3. Smit E, Garcia-Palmieri MR, Figueroa NR, McGee DL, Messina M, Freudenheim JL, et al. Protein and legume intake and prostate cancer mortality in Puerto Rican men. Hnuc. 2007;58(2):146-52.

4. Bates CJ, Mansoor MA, Pentieva KD, Hamer M, Mishra GD. Biochemical risk indices, including plasma homocysteine, that prospectively predict mortality in older British people: the National Diet and Nutrition Survey of People Aged 65 Years and Over. British journal of nutrition. 2010;104(6):893-9.

5. Levine ME, Suarez JA, Brandhorst S, Balasubramanian P, Cheng C-W, Madia F, et al. Low protein intake is associated with a major reduction in IGF-1, cancer, and overall mortality in the 65 and younger but not older population. Cell metabolism. 2014;19(3):407-17.

6. Song M, Fung TT, Hu FB, Willett WC, Longo VD, Chan AT, et al. Association of animal and plant protein intake with all-cause and cause-specific mortality. JAMA internal medicine. 2016;176(10):1453-63.

7. Tharrey M, Mariotti F, Mashchak A, Barbillon P, Delattre M, Fraser GE. Patterns of plant and animal protein intake are strongly associated with cardiovascular mortality: the Adventist Health Study-2 cohort. International journal of epidemiology. 2018;47(5):1603-12.

8. Kurihara A, Okamura T, Sugiyama D, Higashiyama A, Watanabe M, Okuda N, et al. Vegetable Protein Intake was Inversely Associated with Cardiovascular Mortality in A 15-Year Follow-Up Study of A General Japanese Population. Journal of atherosclerosis and thrombosis. 2018:44172.

9. Virtanen HEK, Voutilainen S, Koskinen TT, Mursu J, Kokko P, Ylilauri M, et al. Dietary proteins and protein sources and risk of death: the Kuopio Ischaemic Heart Disease Risk Factor Study. The American journal of clinical nutrition. 2019;109(5):1462-71.

10. Budhathoki S, Sawada N, Iwasaki M, Yamaji T, Goto A, Kotemori A, et al. Association of Animal and Plant Protein Intake With All-Cause and Cause-Specific Mortality in a Japanese Cohort. 2019.
