# Supplementary material for: Dietary protein intake and all-cause and cause-specific mortality: results from the Rotterdam Study and a meta-analysis of prospective cohort studies
Source: Eur J Epidemiol. 2020 Feb 19;35(5):411–29. doi: 10.1007/s10654-020-00607-6 (PMC7250948; doi:10.1007/s10654-020-00607-6)
Supplement: Supplementary file 2 — Supplementary material 2 (PPTX 447 kb) [file 10654_2020_607_MOESM2_ESM.pptx]

## Slide 1
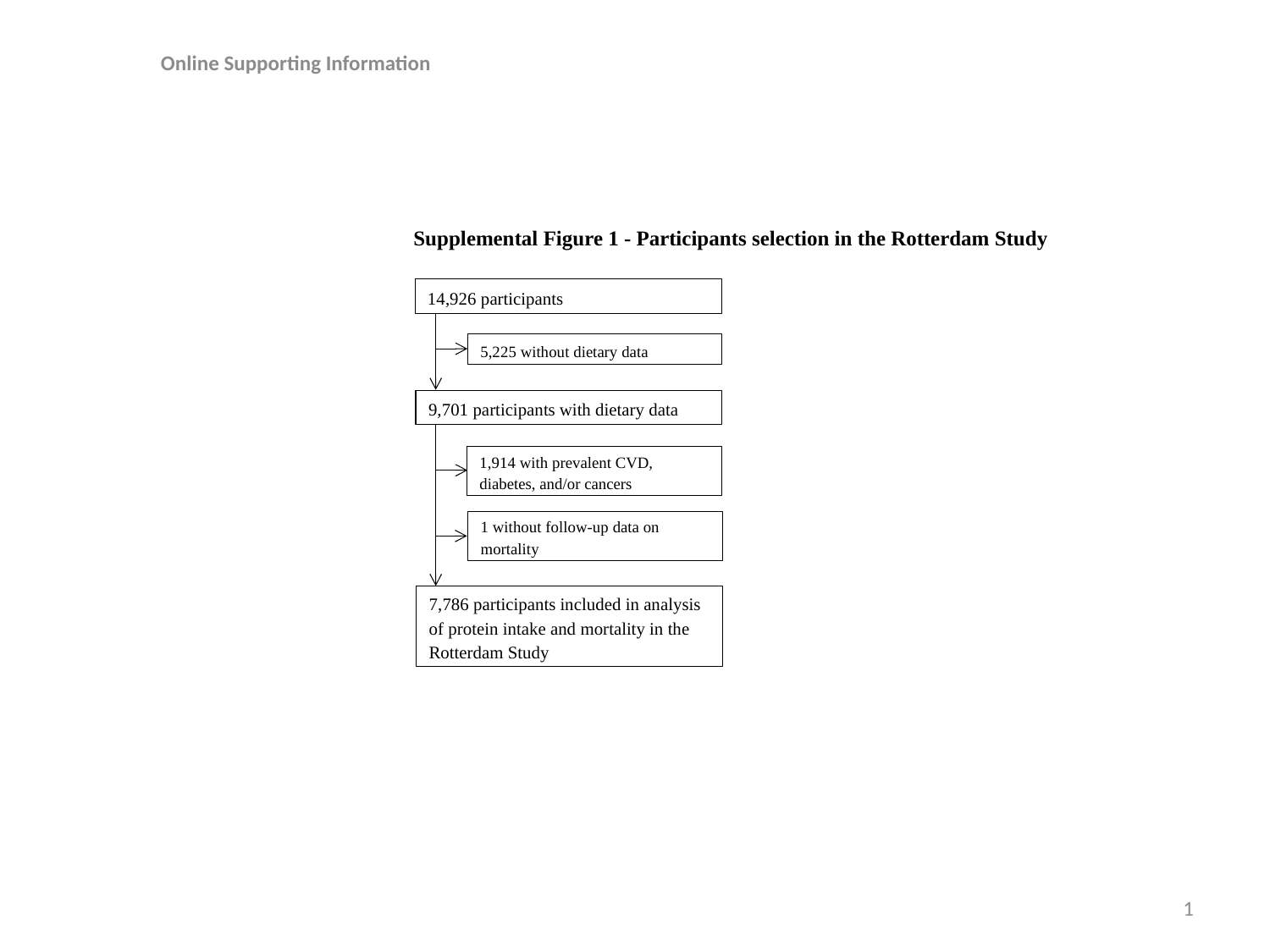

Online Supporting Information
Supplemental Figure 1 - Participants selection in the Rotterdam Study
14,926 participants
5,225 without dietary data
9,701 participants with dietary data
1,914 with prevalent CVD, diabetes, and/or cancers
1 without follow-up data on mortality
7,786 participants included in analysis of protein intake and mortality in the Rotterdam Study
1

## Slide 2
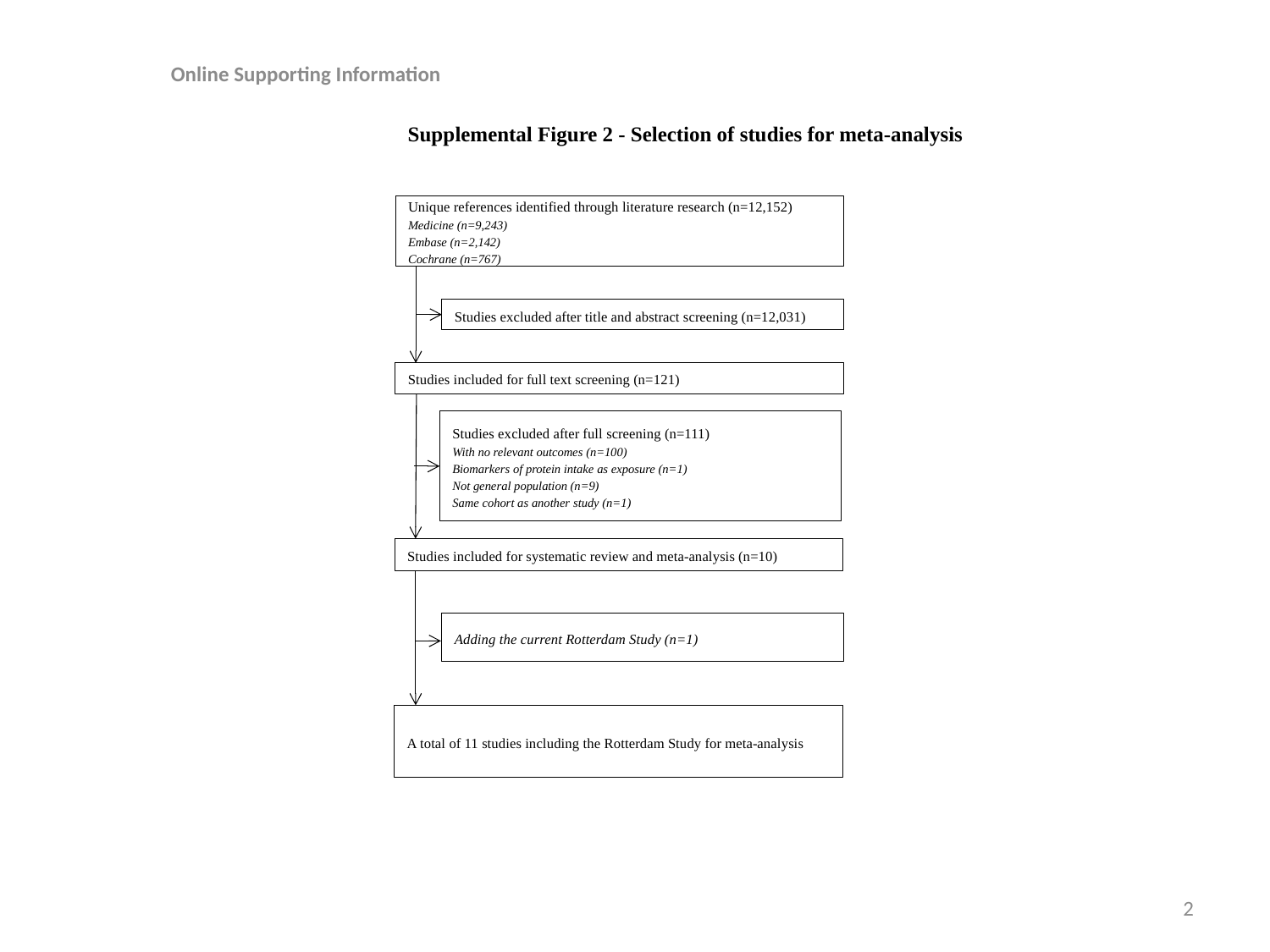

Online Supporting Information
Supplemental Figure 2 - Selection of studies for meta-analysis
Unique references identified through literature research (n=12,152)
Medicine (n=9,243)
Embase (n=2,142)
Cochrane (n=767)
Studies excluded after title and abstract screening (n=12,031)
Studies included for full text screening (n=121)
Studies excluded after full screening (n=111)
With no relevant outcomes (n=100)
Biomarkers of protein intake as exposure (n=1)
Not general population (n=9)
Same cohort as another study (n=1)
Studies included for systematic review and meta-analysis (n=10)
Adding the current Rotterdam Study (n=1)
A total of 11 studies including the Rotterdam Study for meta-analysis
2

## Slide 3
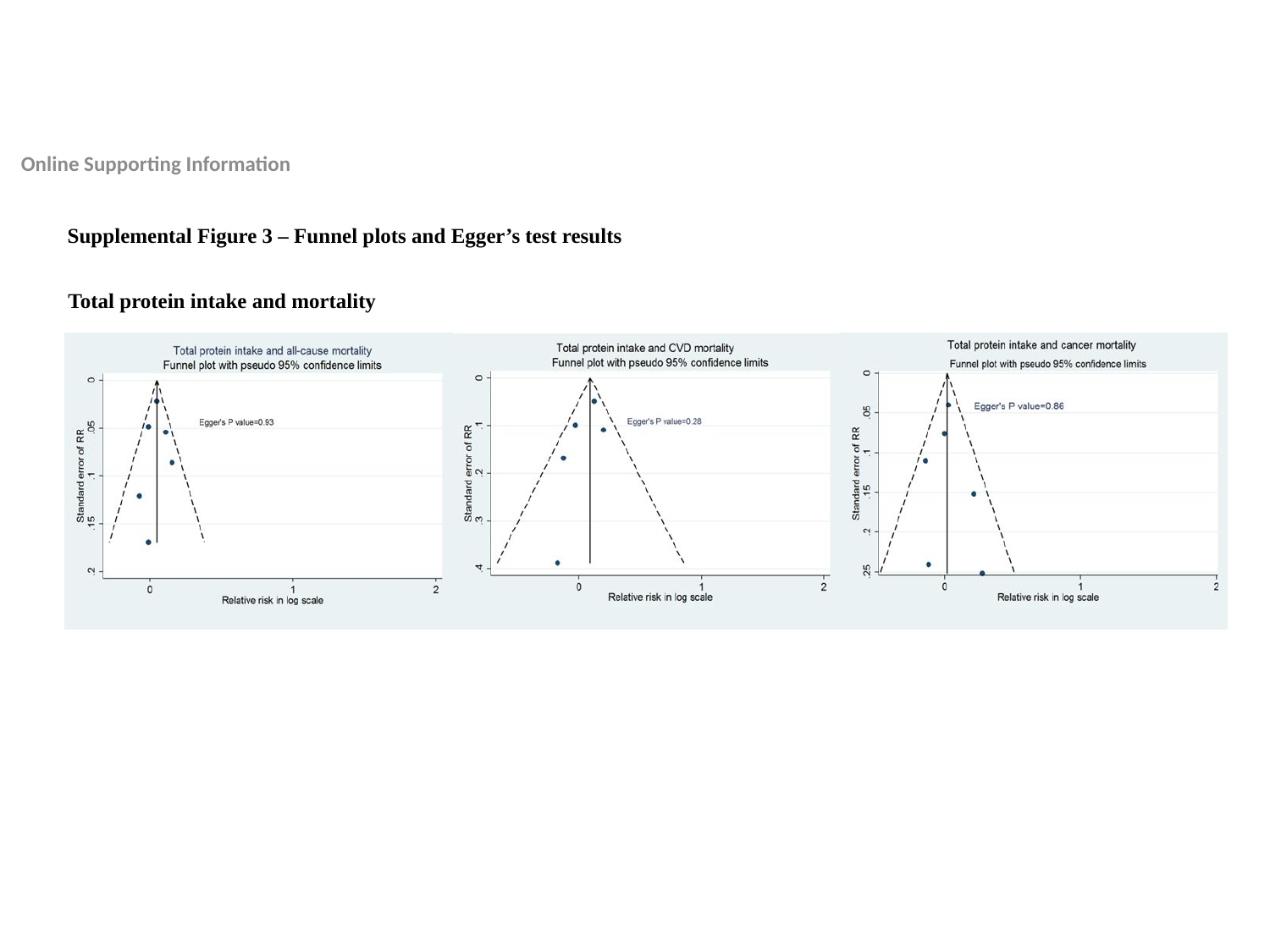

Online Supporting Information
Supplemental Figure 3 – Funnel plots and Egger’s test results
Total protein intake and mortality
3

## Slide 4
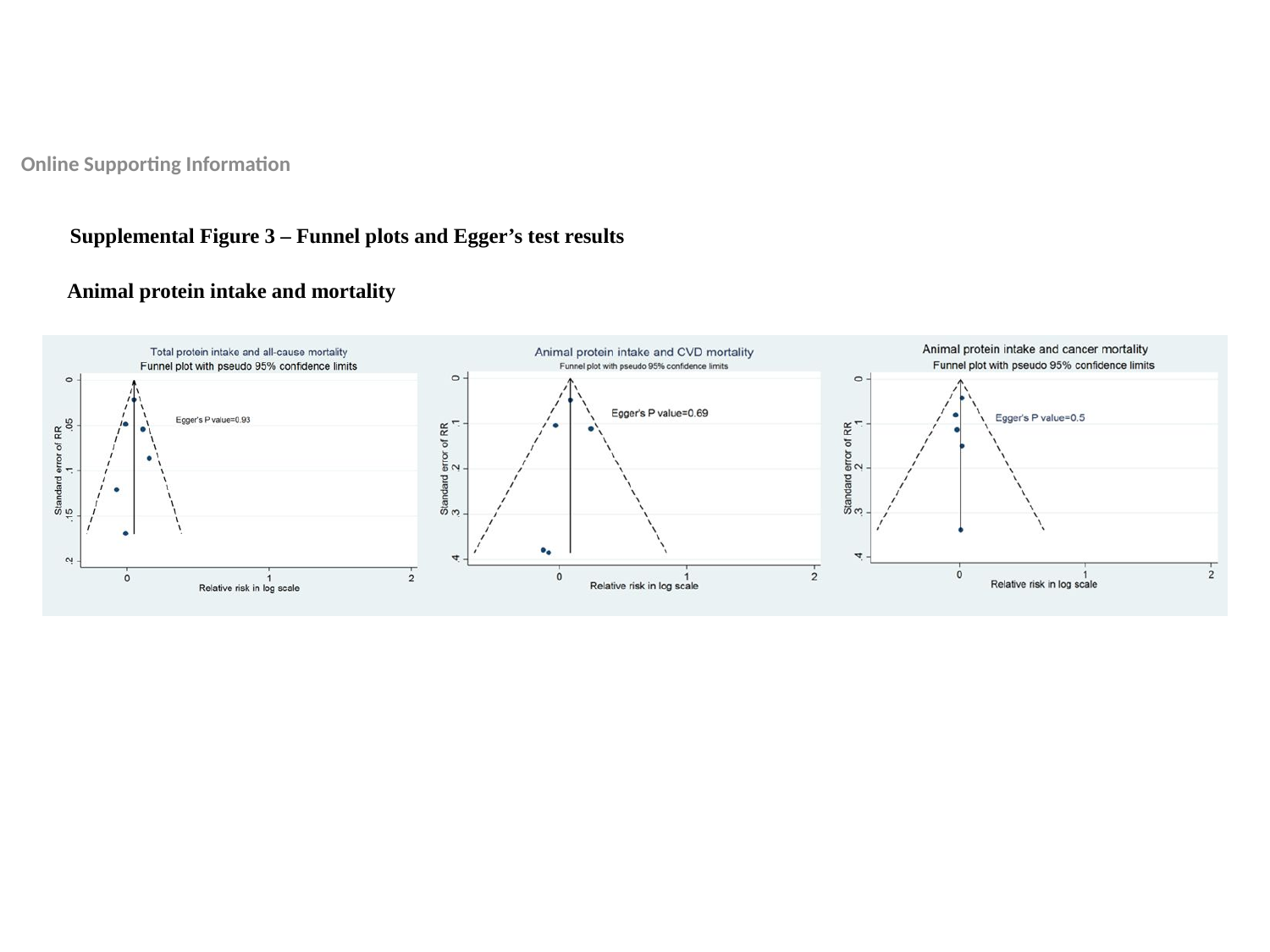

Online Supporting Information
Supplemental Figure 3 – Funnel plots and Egger’s test results
Animal protein intake and mortality
4

## Slide 5
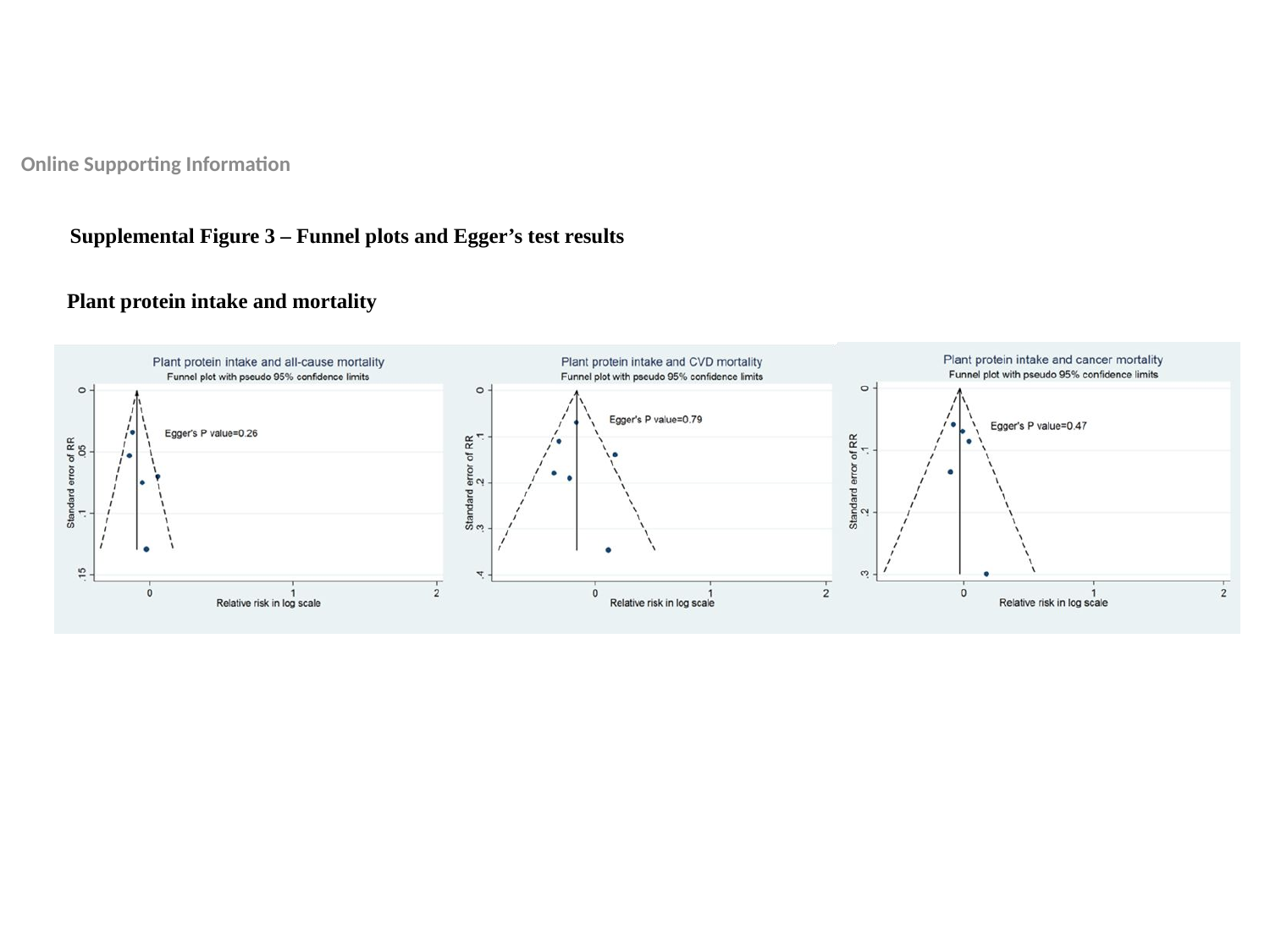

Online Supporting Information
Supplemental Figure 3 – Funnel plots and Egger’s test results
Plant protein intake and mortality
5
